# Caveolin-1 scaffolding domain peptide abrogates autophagy dysregulation in pulmonary fibrosis

**DOI:** 10.1038/s41598-022-14832-4

**Published:** 2022-06-30

**Authors:** Shalini Venkatesan, Liang Fan, Hua Tang, Nagarjun V. Konduru, Sreerama Shetty

**Affiliations:** Texas Lung Injury Institute, Department of Medicine, University of Texas Health Science Center at Tyler, 11937 US Highway 271, Tyler, TX 75708 USA

**Keywords:** Drug discovery, Diseases, Medical research, Pathogenesis

## Abstract

Idiopathic pulmonary fibrosis (IPF) is the most common and fatal form of interstitial lung disease. IPF is characterized by irreversible scarring of the lungs leading to lung function decline. Although the etiology remains poorly understood, dysregulated autophagy in alveolar-epithelial cells (AECs) together with interplay between apoptotic-AECs and proliferative-myofibroblasts have been strongly implicated in IPF pathogenesis. Recent studies have revealed that a caveolin-1-derived 7-mer peptide, CSP7, mitigates established PF at least in part by improving AEC viability. In the present study, we aimed to determine whether and how CSP7 regulates autophagy in fibrotic-lung AECs. We found that p53 and autophagic proteins were markedly upregulated in AECs from mice with single/multi-doses of bleomycin—or silica-induced PF. This was abolished following treatment of PF-mice with CSP7. Further, CSP7 abrogated silica- or bleomycin-induced p53 and autophagy proteins in AECs. Immunoprecipitation further revealed that CSP7 abolishes the interaction of caveolin-1 with LC3BII and p62 in AECs. AEC-specific p53-knockout mice resisted silica- or bleomycin-induced changes in autophagy proteins, or CSP7 treatment. Our findings provide a novel mechanism by which CSP7 inhibits dysregulated autophagy in injured AECs and mitigates existing PF. These results affirm the potential of CSP7 for treating established PF, including IPF and silicosis.

## Introduction

Idiopathic Pulmonary Fibrosis (IPF) is a chronic, life-threatening interstitial lung disease characterized by progressive lung scarring and a key histological hallmark of usual interstitial pneumonia (UIP)^1,2^. IPF affects approximately 3 million people worldwide and 130,000 people have been diagnosed with IPF in the United States (US)^1^. In the US, the annual incidence of IPF in adults (65 + years) is reported to be 93.7 cases per 100,000 person-years^3^. The incidence of IPF is higher in men than women and increases with age^1,2^. The median survival of patients with IPF is approximately 3 to 5 years. Clinical manifestations of IPF include exertional dyspnea, chronic dry cough and/or Velcro-like crackles on examination^4^. Although FDA approved Pirfenidone and Nintedanib for the treatment of IPF, these drugs only slow the disease progression but are not curative^1,4^. Consequently, there is considerable interest to develop better therapeutics for treating patients with IPF.

With unknown etiology, the pathogenesis of IPF is characterized by chronic lung injury, replicative senescence, and apoptosis of type II alveolar epithelial cell (AECs) leading to loss of epithelial regeneration. This results in release of pro-fibrotic cytokines that induce migration, proliferation, and activation of fibroblasts and myofibroblasts that resist apoptosis and secrete extracellular matrix proteins, leading to progressive interstitial fibrosis^1,4^. Autophagy is an evolutionarily conserved lysosome based degradative process that plays a pivotal role in regulation of apoptosis^5–7^. Macroautophagy (hereafter simply referred to as autophagy) is the most-well studied form of autophagy which involves formation of double-membranous vesicular structure (encapsulating the cytoplasmic cargo) called autophagosome that eventually fuses with lysosome to degrade its contents^5,8^. Autophagy is a highly dynamic process consisting of well-orchestrated discrete stages controlled by at least 34 known autophagy (ATG) genes^5^. Lipidation of microtubule-associated protein 1 light chain-3 (LC3), a homologue of ATG8 is a characteristic feature of autophagosome formation^5,6^. Conjugation of ATG12-ATG5 with ATG16L1 are required for autophagosome assembly^7^. Sequestosome 1 (SQSTM1/p62) acts as a cargo receptor protein that is capable of binding both polyubiquitinated substrate and LC3^5,9^. Accumulating evidence based on studies reporting the existence of dysregulated autophagy in human IPF and mouse models of PF suggests that aberrant autophagy triggers AEC apoptosis and promotes myofibroblast differentiation^6–11^.

Caveolae are 50–100 nm flask-shaped invaginations of the plasma membrane that play critical role in vesicular trafficking, lipid metabolism and compartmentalization of specific signaling cascades. Caveolins (Cav) are the 22–24 kDa caveolae resident structural and scaffolding proteins. Among the three known Cav isoforms, Cav1 is the most extensively studied and is regarded as the principal component of the caveolae^12,13^. Cav1 is ubiquitously expressed in differentiated cells such as fibroblasts, AECs and endothelial cells^13–18^. Cav1 acts as a molecular hub that integrates and regulates multiple signaling pathways including the transforming growth factor-beta (TGF-β) signaling^12^. Cav1^-/-^ mice resists bleomycin (BLM)-induced senescence and apoptosis of epithelial cells and are protected against development of PF^14^. Thus, Cav1 could serve as a potential therapeutic target for the treatment of PF^13,15^.

The interaction of Cav1 and signaling molecules is believed to be mediated by Cav1 scaffolding domain corresponding to amino acids 82–101 present at the N-terminal region of Cav1^12^. We and others have demonstrated the antifibrotic effects of 20-mer Cav1 scaffolding domain peptide (CSP)^16–19^. We have also shown that CSP prevents AECs apoptosis and activation of myofibroblasts and fibrotic lung fibroblasts (fLfs) through regulation of p53 signaling^16–18^. Moreover, we recently reported that systemic (or) local delivery of a 7-mer deletion fragment of CSP (CSP7) resolves PF at a comparable efficiency to CSP and was shown to attenuate apoptosis of AECs in three mouse models with established PF^20^. Similarly, treatment of human end-stage IPF lung tissues with CSP7 ex vivo inhibited p53 and active caspase-3 in AECs isolated from the disease lung tissues, and pro-fibrogenic proteins in lung tissue homogenates^20^. The focus of the current study was to investigate the underlying molecular mechanisms by which CSP7 prevents dysregulation of autophagy in AECs representing different exposure-induced PF mouse models. Further, we also sought to know whether CSP7-mediated inhibition of autophagy in AECs involved the interaction of Cav1 with autophagy proteins.

## Results

### CSP7 mitigates silica-induced lung fibrosis in mice

**Figure 1:**
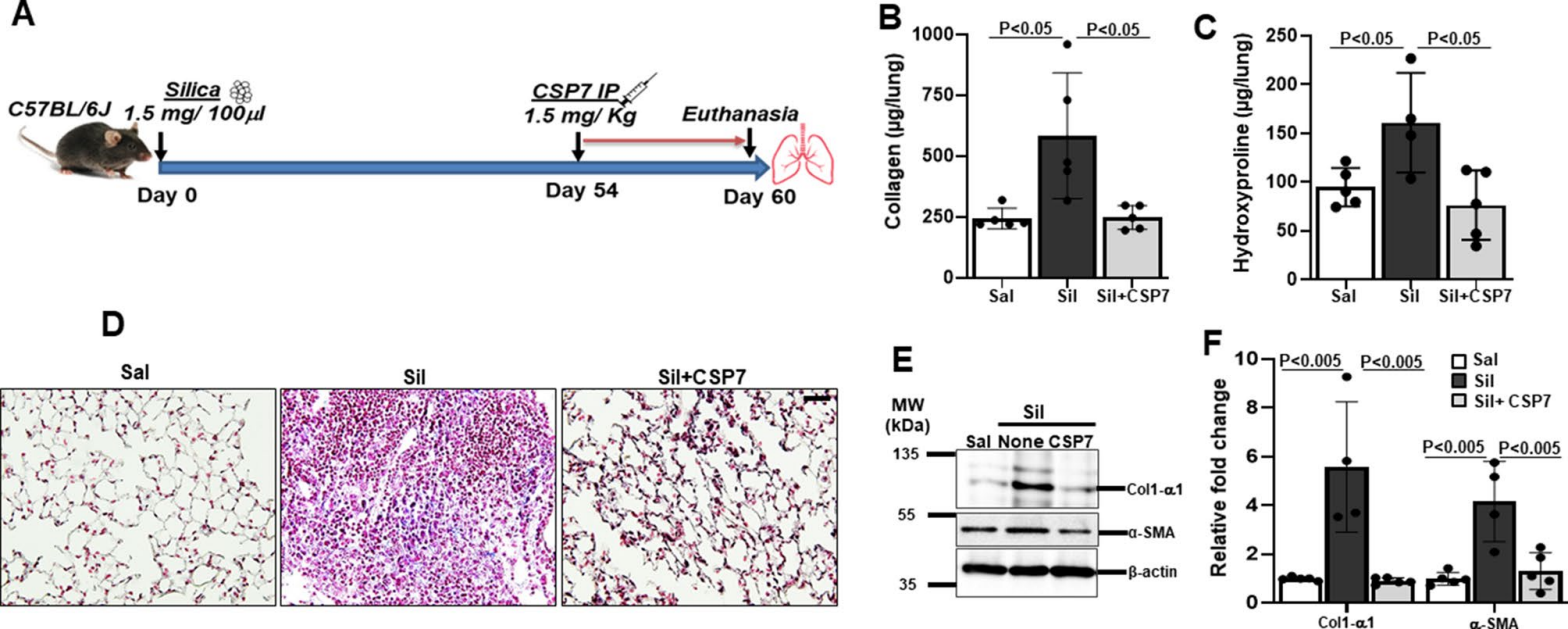
CSP7 mitigates silica-induced existing PF. (**A**) Schematic diagram showing the experimental design. (**B**) Bar graphs showing inhibition of total lung soluble collagen and (**C**) hydroxyproline contents in mice (n = 5–7 per group) with silica-induced PF and subjected to CSP7 treatment for last 7 days as in Fig. 1A. (**D**) Representative trichrome stained lung tissue sections showing increased Col1 deposition in silica instilled WT mice and its reversal by CSP7 treatment. Scale bar: 20 μm. (**E**) Representative western blot images of lung homogenates showing marked increase in Col1 and α-SMA protein expression in silica treated WT mice, which is attenuated after CSP7 treatment. β-actin serves as loading control. MW: Molecular weight. kDa: Kilodalton. (**F**) Bar graph depicting decreased expression of Col1 and α-SMA mRNA in lung homogenates of CSP7 treated WT mice (n = 5) exposed to silica. Each experiment was repeated at least 2–3 times and data is presented as mean + SD and *p* values were obtained by one-way Anova with Turkey’s multiple comparison test and log-rank tests, respectively.

Our experimental investigations to test the effects of CSP7 on autophagy utilized an established murine model of lung fibrosis wherein fibrosis was induced by intratracheal instillation of crystalline silica (1.5 mg/100 μl) (Fig. 1A). We observed development of fibrotic nodules in lung tissues of mice 60 days post-exposure to silica (Figure S1), a histopathological feature that typifies the silica-induced PF. Although the extent of fibrosis was less dense than that induced by BLM treatment, quantitative analyses of Col1 (Fig. 1B) and hydroxyproline (Fig. 1C) content, surrogates for assessment of total collagen levels, were found to be significantly increased in the whole lung homogenates of silica-exposed mice. Further, trichrome staining of lung sections also exhibited prominently increased Col and other extracellular matrix protein deposition (Fig. 1D). The expression of Col1 and α-SMA protein (Fig. 1E) and mRNA (Fig. 1F) were also prominently upregulated in the lungs of mice exposed to silica, thus biochemically confirming the development of PF. As evident in multiple mouse models of PF^20^, we observed intraperitoneal (IP) administration of CSP7 (1.5 mg/kg) once daily from day 54 through day 60 post-silica exposure significantly reduced soluble Col and total hydroxyproline (Fig. 1B–C). This was also independently confirmed by Masson’s Trichrome staining of lung sections (Fig. 1D), analysis of Col1 and α-SMA protein (Fig. 1E) and mRNA (Fig. 1F), suggesting mitigation of silica-induced PF by CSP7 treatment.

### CSP7 inhibits autophagy dysregulation in silica-induced lung fibrosis in mice

**Figure 2:**
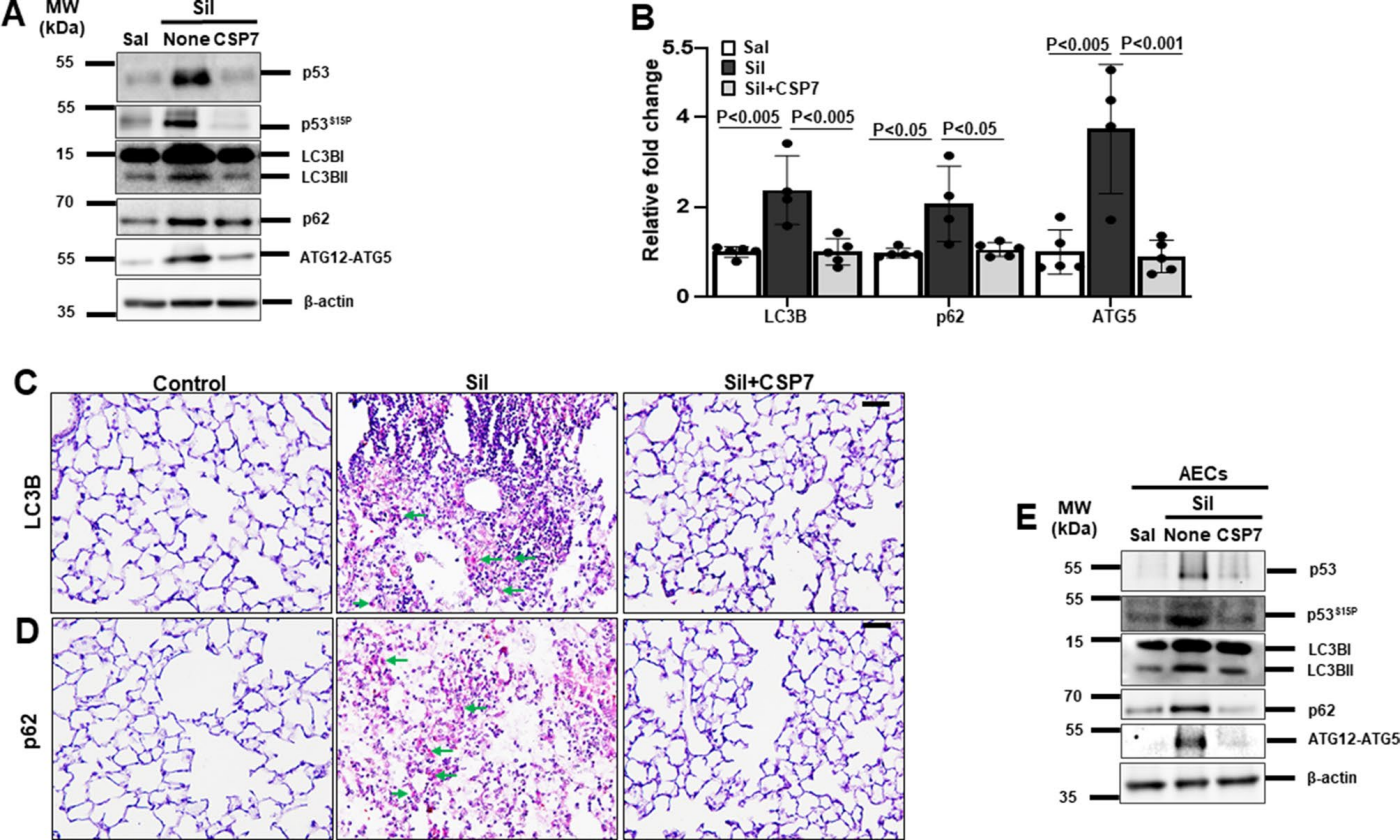
Abrogation of aberrant autophagy by CSP7 in mice with silica-induced PF. (**A**) Representative Western blot images showing the expression of p53, p53 phosphorylation at serine 15 residue (p53^S15P^), lipidated LC3B (LC3BII), p62 and ATG12-ATG5 complex in lung homogenates of naïve WT mice, and WT mice (n = 5–7) with silica-induced PF left untreated or treated with CSP7 as described in Fig. 1A. β-actin serves as loading control. MW: Molecular weight. kDa: Kilodalton. (**B**) Bar graph showing decreased LC3B, p62 and ATG5 mRNA in the lung homogenates of silica-exposed WT mice treated with CSP7. Representative immunohistochemistry images showing inhibition of (**C**) LC3B and (**D**) p62 expression by CSP7 in lungs of WT mice exposed to silica. Scale bar: 20 μm. (**E**) Representative immunoblot images showing inhibition of p53, p53^S15P^, LC3BII, p62 and ATG12-ATG5 expression in AECs isolated from silica exposed WT mice treated with CSP7 in vivo*.* β-actin serves as loading control. MW: Molecular weight. kDa: Kilodalton. Each experiment was repeated at least 2–3 times and data is presented as mean + SD and *p* values were obtained by one-way Anova with Turkey’s multiple comparison test and log-rank tests, respectively.

As aberrant autophagy plays a pivotal role in apoptosis of AECs and development of PF, we therefore explored the potential of CSP7 in regulating autophagy in silica-induced PF mouse model. Since conversion of LC3BI (non-lipidated) to LC3BII (lipidated) is a characteristic feature of autophagy and autophagosome formation, we evaluated the lipidation of LC3B in the whole lung homogenates of silica-exposed mice with PF that were either left untreated or treated with CSP7. As anticipated and reported^21–23^, we found a significant increase in the lipidation of LCBII in mice with silica-induced PF compared to saline treated controls (Fig. 2A). The levels of lipidated LCBII were markedly reduced in silica-exposed mice subjected to CSP7 treatment (Fig. 2A). In addition, mRNA level of LC3B was also found to be significantly elevated in the lungs of silica-exposed mice that was inhibited following CSP7 treatment (Fig. 2B). We analysed the expression of protein and mRNA of autophagy substrate and cargo receptor protein, p62/SQSTM1, both of which were markedly increased in the lungs of silica-exposed mice and reduced upon CSP7 treatment (Fig. 2A–B). Immunohistochemical (IHC) analysis of lung tissue sections from mice exposed to silica also showed decreased LC3B and p62 expression post-treatment with CSP7, which was otherwise induced by silica exposure (Fig. 2C–D). Similarly, the expression of ATG5 was also increased at the transcriptional and translational level in the lungs of silica-exposed mice in contrast to those treated with CSP7 (Fig. 2A–B). Corroborating the findings from in vivo experiments, AECs isolated from silica-exposed mice demonstrated increased LCB lipidation (LC3BII) and increased expression of p62 and ATG5; and these changes were reversed following treatment with CSP7 in silica-exposed mice (Fig. 2E). Our previous works^17,18,20^ show that CSP7 inhibits AEC apoptosis by downregulating p53 and serine 15 phosphorylation of p53 (p53^S15P^), and others have reported that p53 directly regulates autophagy^23–25^. Therefore, we sought to investigate whether CSP7 acts via p53 to regulate autophagy and found that CSP7 suppressed silica-induced p53 and p53^S15P^ expression in AECs (Fig. 2A, E).

### Abrogation of aberrant autophagy by CSP7 in 1X-BLM-induced lung fibrosis in mice

**Figure 3:**
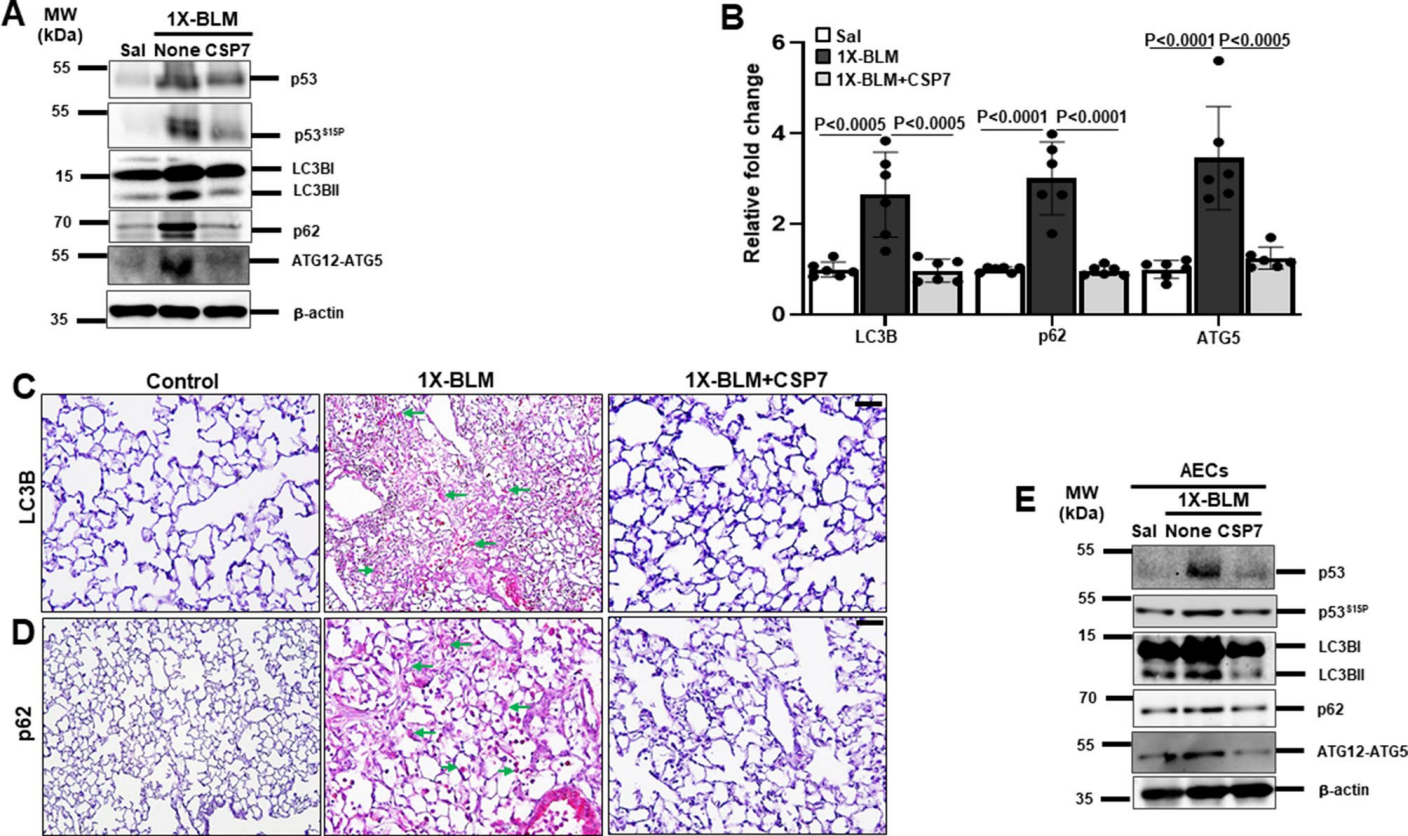
CSP7 inhibits autophagy dysregulation in 1X-BLM-induced PF. (**A**) Representative Western blot images showing expression of p53, p53^S15P^, LC3BII, p62 and ATG12-ATG5 in lung homogenates of 1X-BLM-PF WT mice (n = 6–7 per group) treated with CSP7. β-actin serves as loading control. MW: Molecular weight. kDa: Kilodalton. (**B**) Bar graph showing decreased expression of LC3B, p62 and ATG5 mRNA in the lungs of CSP7 treated 1X-BLM-PF mice (n = 6). Representative immunohistochemistry images showing reduced expression of (**C**) LC3B and (**D**) p62 by CSP7 in the lung tissue sections of CSP7 treated 1X-BLM-PF WT mice. Scale bar: 20 μm. (**E**) Representative immunoblot images showing p53, p53^S15P^, LC3BII, p62 and ATG12-ATG5 expression in AECs isolated from WT mice with 1X-BLM-induced PF and exposed to CSP7. β-actin serves as loading control. MW: Molecular weight. kDa: Kilodalton. Each experiment was repeated at least 2 times and data is presented as mean + SD and *p* values were obtained by one-way Anova with Turkey’s multiple comparison test and log-rank tests, respectively.

To further validate our findings, we decided to confirm the effect of CSP7 on autophagy regulation in single- (1X) and repeat-dose (8X) BLM-induced murine model with existing PF. For 1X BLM-exposed mice that received a one-time intra-tracheal instillation of BLM (2U/Kg)^20^, CSP7 (1.5 mg/kg) was administered intraperitoneally once daily for 1 week starting with day 15. To confirm the development of lung fibrosis in this model, we analysed the expression of Col1 and α-SMA at the level of protein and mRNA and found both were significantly increased; and CSP7 treatment inhibited its upregulation (Figure S2) as demonstrated earlier^20^. Next, we repeated the experiments that were performed in mouse model of silica-induced PF (Fig. 2) with 1X-BLM model. Western blot analysis of lung homogenate of 1X-BLM-PF mice revealed remarkable increase in p53, p53^S15P^, LC3BII, p62 and ATG12-ATG5; and CSP7 treatment inhibited these parameters (Fig. 3A). CSP7 treatment also significantly reduced the expression of LC3B, p62 and ATG5 mRNA that were otherwise upregulated in mice with PF induced by 1X-BLM (Fig. 3B). Consistent with these findings, IHC analysis demonstrated marked decrease in LC3B and p62 antigen levels in lung sections of 1X-BLM-PF mice treated with CSP7. Interestingly, the levels of LC3B and p62 antigen in CSP7 treated mice were found to be similar to the levels observed in saline treated control mice but different from the levels seen in mice with BLM-PF that were left untreated (Fig. 3C–D). Immunoblotting of isolated AECs from these mice exhibited similar pattern of protein expression as seen with their corresponding lung homogenates (Fig. 3E), confirming the ability of CSP7 to regulate autophagy in injured AECs besides mitigation of 1X-BLM-induced PF.

### Inhibition of altered autophagy by CSP7 in mice with 8X-BLM-induced PF

**Figure 4:**
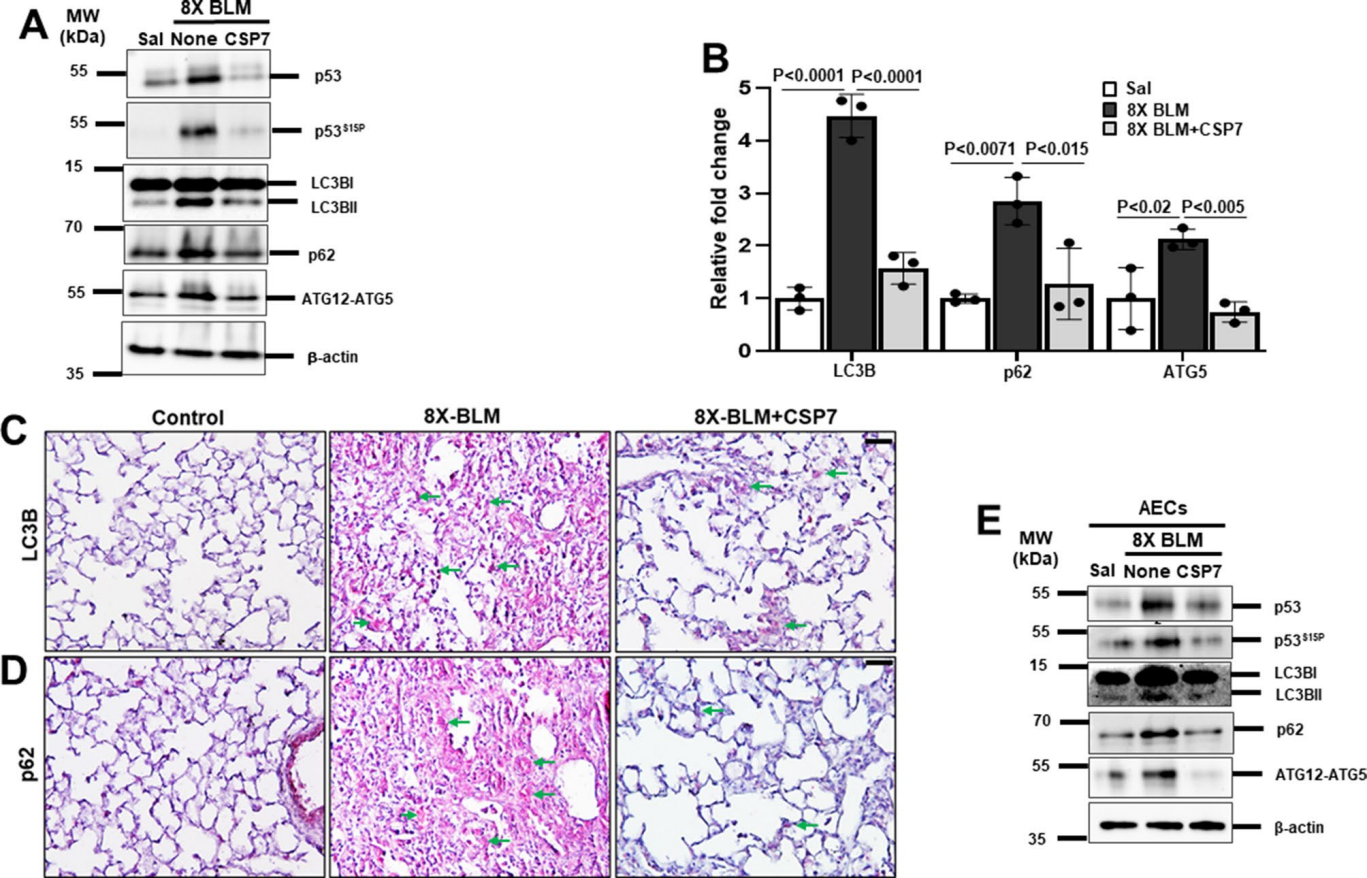
CSP7 mitigates autophagy dysregulation in 8X-BLM-induced PF. (**A**) Representative western blot images showing the expression of p53, p53^S15P^, LC3BII, p62 and ATG12-ATG5 in lung homogenates of CSP7 treated WT mice (n = 5) with 8X-BLM-induced existing PF. β-actin serves as loading control. MW: Molecular weight. kDa: Kilodalton. (**B**) Bar graph showing increased expression of LC3B, p62 and ATG5 mRNA in the lungs of 8X-BLM-PF WT mice, which is inhibited upon CSP7 treatment. Representative immunohistochemistry images showing inhibition of (**C**) LC3B and (**D**) p62 expression by CSP7 in lungs of WT mice with 8X-BLM-PF. Scale bar: 20 μm. (**E**) Representative immunoblot images showing inhibition of p53, p53^S15P^, LC3BII, p62 and ATG12-ATG5 expression in AECs isolated from WT mice with 8X-BLM-induced existing PF treated with CSP7 in vivo. β-actin serves as loading control. MW: Molecular weight. kDa: Kilodalton. Each experiment was repeated at least 2–3 times and data is presented as mean + SD and *p* values were obtained by one-way Anova with Turkey’s multiple comparison test and log-rank tests, respectively.

Mouse model of 8X-BLM-induced PF was established and CSP7 (1.5 mg/kg) was administered by IP injection daily for 2 weeks after the final dose of BLM as we described^20^. Consistent with our findings in silica- or 1X BLM-induced mouse models of PF, we found that CSP7 decreased total lung Col1 and α-SMA protein and mRNA, suggesting resolution of chronic/repetitive BLM-induced PF in mice. Similarly, the expression of proteins such as p53, p53^S15P^, LC3BII, p62 and ATG12-ATG5 in 8X-BLM treated lungs of mice were reduced to normal levels seen in control mice after CSP7 treatment (Fig. 4A). On similar lines, increased mRNA levels of LC3B, p62 and ATG5 observed in the whole lung homogenates from 8X-BLM mice were downregulated by CSP7 treatment (Fig. 4B). IHC analysis of lung tissue sections of CSP7 treated 8X-BLM mice also showed decreased LC3B and p62 expression compared to those from 8X-BLM left untreated (Fig. 4C–D). Further, CSP7 treatment reduced the elevated p53, p53^S15P^, LC3BII, p62 and ATG12-ATG5 levels found in AECs isolated from 8X-BLM-PF mice to near-normal (Fig. 4E). Collectively, our findings show that CSP7 treatment attenuates BLM-induced lung fibrosis (Figure S3) and that the antifibrotic effects of CSP7 partly involves mitigation of aberrant autophagy in AECs.

### CSP7 prevents silica- and BLM-induced autophagy dysregulation in hAECs in vitro

**Figure 5:**
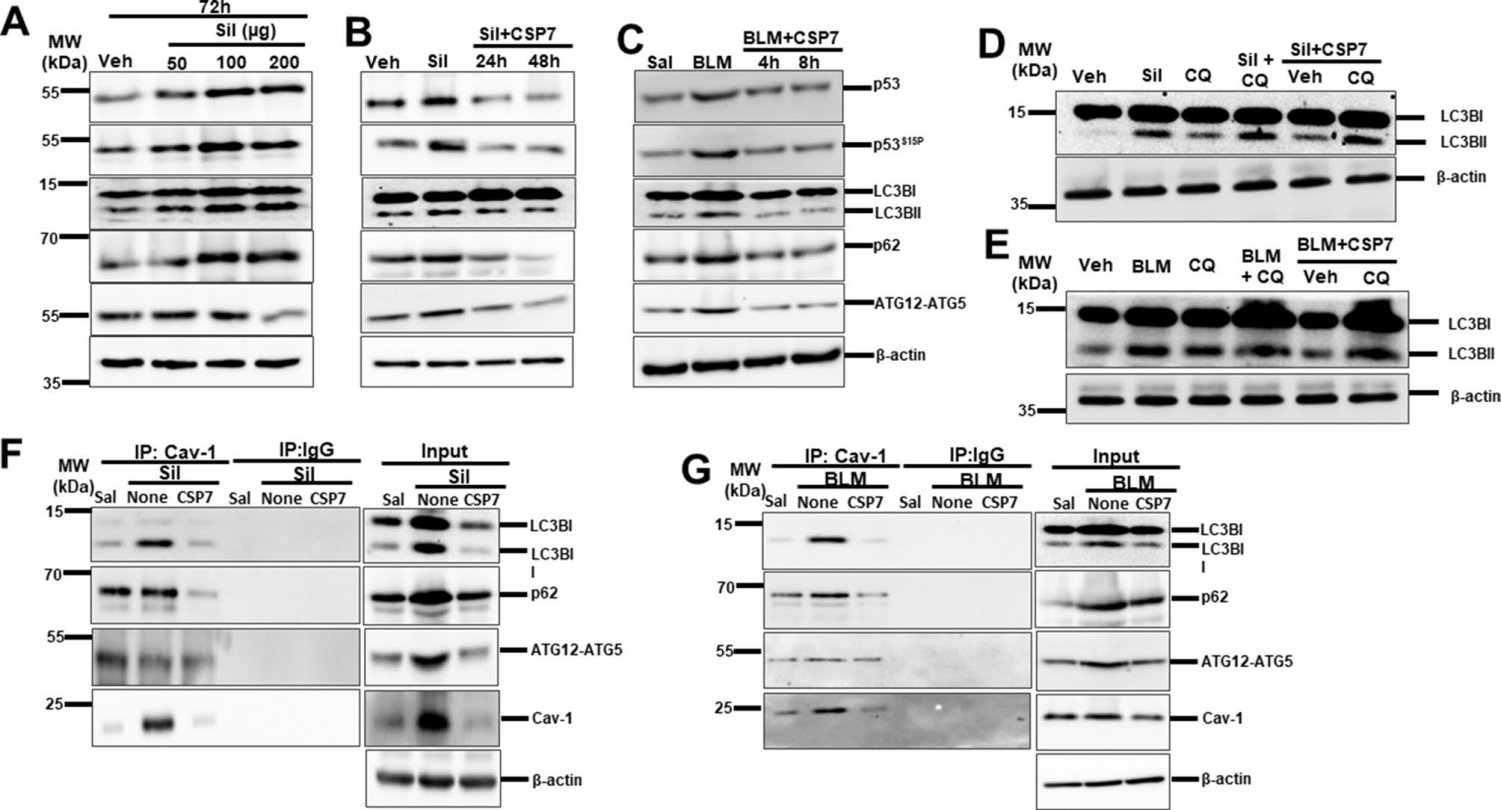
CSP7 hinders silica- and BLM-induced autophagy in hAECs in vitro. (**A**) Representative immunoblot images showing concentration dependent expression of p53, p53^S15P^, LC3BII, p62 and ATG12-ATG5 in hAECs exposed silica for 72 h. (**B**) Representative immunoblot showing decrease in p53, p53^S15P^, LC3BII, p62 and ATG12-ATG5 expression in hAECs exposed to silica and treated with CSP7 for 24 and 48 h. (**C**) Representative Western blot images showing inhibition of p53, p53^S15P^, LC3BII, p62 and ATG12-ATG5 in hAECs exposed to BLM followed by CSP7 for 4 and 8 h. Representative immunoblot images showing autophagy flux analysis in hAECs (**D**) silica and (**E**) BLM exposure injury. Representative Western blot images showing immunoprecipitation (IP) of LC3B and p62 by Cav1 in hAECs exposed to (**F**) silica and (**G**) BLM, which is abrogated by CSP7 treatment. Immunoblots of IP with non-specific IgG and inputs of the corresponding lysates of hAECs serve as controls. β-actin serves as loading control. MW: Molecular weight. kDa: Kilodalton. Each experiment was repeated at least 2 times with similar results.

To test whether CSP7 prevents silica- and BLM-induced aberrant autophagy in hAECs, cells were treated with different concentrations of silica for 72 h in vitro as we previously described^26^. We observed a dose-dependent increase in the expression of p53, p53^S15P^, LC3BII, p62 and ATG12-ATG5 protein with maximum induction at 100 µg/mL of silica treatment (Fig. 5A). Treatment of hAECs with CSP7 (20 μM) at 24 or 48 h post-silica injury drastically reduced the expression of p53, p53^S15P^, LC3BII, p62 and ATG12-ATG5 proteins (Fig. 5B). Similarly, treatment of hAECs with CSP7 (10 μM) 4 or 8 h after BLM (40 μg/ml)^17^ decreased p53, p53^S15P^, LC3BII, p62 and ATG12-ATG5 expression 24 h later (Fig. 5C). Since increased LC3BII level does not directly indicate change in autophagy, to determine autophagy flux, we next treated silica exposed hAECs with chloroquine (CQ) or vehicle in the presence or absence of CSP7. We observed a significant increase in the levels of LC3BII in CQ and silica co-treated hAECs compared to hAECs exposed to silica or CQ alone, indicating an induction of autophagy by silica in hAECs. Further, treatment of silica- and CQ-exposed hAECs with CSP7 also increased LC3BII levels (Fig. 5D), suggesting that CQ-mediated inhibition of autophagy is unaffected by CSP7. Similar results were also noted with co-treatment of CQ and BLM of hAECs (Fig. 5E). Since Cav1 is known to interact with autophagy proteins^27–29^, we sought how this interaction is altered during AEC injury and whether it is affected by CSP7 treatment. Consistent with elevated expression of Cav1 during silica- or BLM-induced AEC injury^26^, we found increased interaction of LC3BII and p62 Cav1 with both in hAECs with silica (Fig. 5F) or BLM (Fig. 5G) injury. These interactions were attenuated by CSP7 without interfering with the interactions between Cav1 and ATG5 in injured hAECs. These results further confirm that CSP7 prevents aberrant autophagy in injured hAECs by inhibiting the interaction of Cav1 with LC3BII and p62.

### CSP7 regulates autophagy via p53 in injured AECs during silica- and 1X-BLM-induced PF

**Figure 6:**
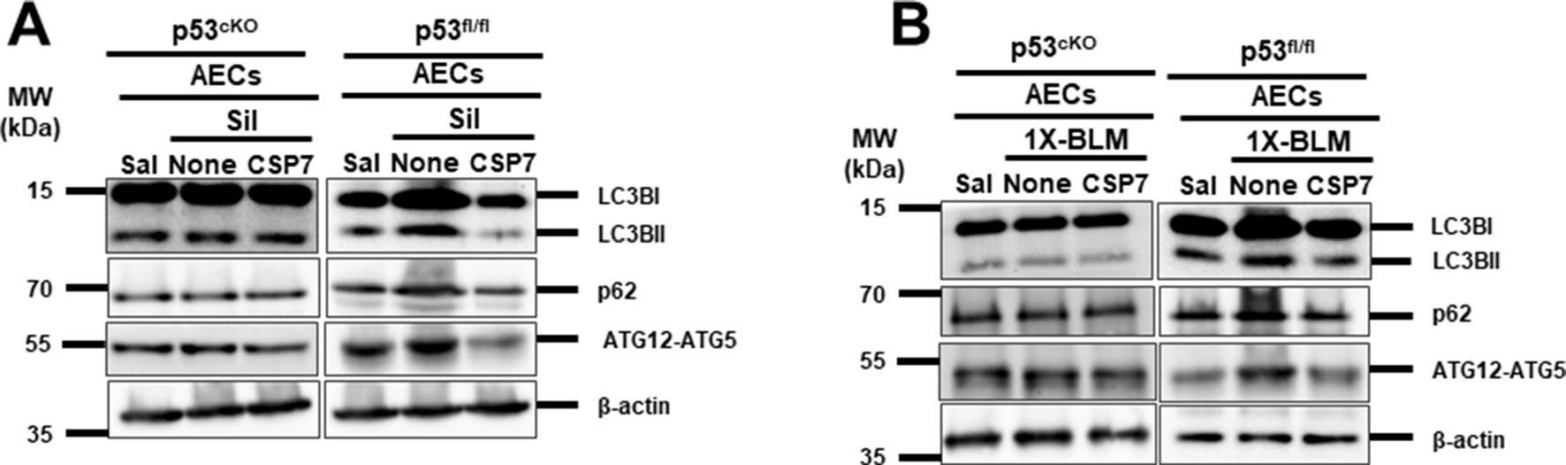
CSP7 regulates autophagy via p53 in AECs of mice with silica- and 1X-BLM-induced PF. (**A**) Representative Western blot images showing lipidation of LC3B (LC3BII) and expression of p62 and ATG12-ATG5 in AECs of p53^cKO^ and p53^fl/fl^ mice (n = 5–6 per group) exposed to silica and post-silica CSP7 treatment. β-actin serves as loading control. (**B**) Representative Western blot images showing the expression of LC3BII, p62 and ATG12-ATG5 in AECs isolated from p53^cKO^ and p53^fl/fl^ mice exposed to 1X-BLM. β-actin serves as loading control. MW: Molecular weight. kDa: Kilodalton. Each experiment was repeated at least 2 times with similar results.

Since we have previously observed increased p53 and p53^S15P^ expression in injured AECs of all three murine models of PF^17,18,20^ and as p53 could directly regulate autophagy^24,25^ we wanted to determine whether CSP7 regulates autophagy via p53. To this end, we treated p53^cKO^ lacking p53 expression in AECs and p53^fl/fl^ mice with 1X-silica or 1X-BLM as described before and the isolated AECs were analysed for the expression levels of autophagic proteins. We noted that in the absence of p53 expression (p53^cKO^ mice *vs* p53^fl/fl^ mice) both 1X-silica and 1X-BLM failed to induce autophagy proteins LC3BII, p62 and ATG5 in AECs, and CSP7 treatment had minimal effect (Fig. 6). These results clearly indicate that p53 plays a central role in regulating autophagy in injured AECs and that CSP7 prevents autophagy dysregulation through inhibition of p53 expression.

## Discussion

In our recent report, we demonstrated that systemic or airway delivery of a 7-mer peptide fragment of Cav1 scaffolding domain, CSP7 effectively resolves different mouse models of existing PF^20^. In line with this, we also wanted to test if CSP7 could resolve silica-induced PF or silicosis in mice. Like IPF, silicosis is also an interstitial lung disease that involves pulmonary inflammation, formation of lung nodules and development of PF. Silicosis is caused by inhalation of crystalline silica or silicon dioxide. Occupational exposure to respirable crystalline silica occurs in a variety of industrial settings^30^. Approximately, 2 million workers in the US are exposed to silica at the workplace^31^. Once established, silicosis continues to develop even after the occupational exposures are stopped^32^. Although preventable by limiting exposure, silicosis currently has no cure^30^. In mice with silica-induced PF, we found that systemically administered CSP7 significantly inhibited the development of PF, which was evident from decreased synthesis and deposition of extracellular matrix proteins like Col1 and α-SMA. This is consistent with earlier reports of CSP/CSP7 mitigating PF through inhibition of AECs apoptosis, fLf activation and expansion^16–20,33^. Besides PF, CSP inhibits development of fibrosis in other organs such as skin, liver, heart, and kidney^34–37^.

Dysregulated autophagy has been reported in different cell types and is involved in the pathogenesis of PF^8–10,21–23^. In agreement with the literature, we found aberrant autophagy in injured AECs of mice with silica- and BLM-induced PF. The expression of autophagy markers, namely LC3BII, p62 and ATG12-ATG5 proteins and their transcripts were markedly increased in injured AECs. Accumulation of autophagy substrate, p62, signifies inhibition of autophagy^8,9,38^, and since upregulation of p62 can also occur at the transcriptional level, increased p62 therefore could no longer serve as a direct indicator of autophagy inhibition^38,39^. However, analysis of autophagy flux further revealed increased autophagy in injured AECs. On the contrary, fewer studies show blockade in autophagy flux in injured AECs leading to the development of PF^10,21,40^. The discrepancy in these above reports could mainly be attributed to the varied cell types used in the respective studies as they respond differently to injury. Further investigations are required to understand whether dysregulation of autophagy observed in injured AECs implies an induction or an inhibition of autophagy. Since it is beyond the scope of the current study, these aspects were not pursued. In addition, the lack of inhibition of LC3B lipidation by CSP7 in CQ treated injured AECs indicates that the mechanism of action of CSP7 might be different from the injury inflicted by CQ or that the concentration of CSP7 used was not sufficient to prevent LC3B lipidation in combined silica or BLM and CQ treated AECs.

We have previously reported that there is increased expression of Cav1 in injured AECs isolated from the lungs of IPF patients and mouse models of established PF, which is attenuated by CSP/CSP7 treatment. In this study, we found competitive inhibition of Cav1 by CSP7 to mitigate autophagy dysregulation in injured AECs of PF models. Existing literature show that Cav1 could differentially regulate autophagy in different disease conditions^41–46^. Cav1 expression correlates directly with the expression of autophagy proteins LC3II, ATG12-ATG5 and Beclin1 in breast cancer cell line following treatment with estradiol^43^. Phosphorylated Cav1 is shown to act as a positive regulator of autophagy in cerebral ischemic injury^42^. Aldosterone induced Cav1 and autophagy is reported to facilitate liver sinusoidal endothelial cell defenestration^44^. Cav1 expression has also been inversely correlated with autophagy in diseases like atherosclerosis and several types of cancers such as osteosarcoma, chronic myelogenous leukemia and hepatocellular carcinoma^45,46^. These studies suggest that Cav1 expression and autophagy regulation are context-specific.

Further, our Co-immunoprecipitation studies revealed increased interaction of Cav1 and autophagy proteins, LC3BII and p62 in injured AECs, suggestive of elevated expression of Cav1 resulting from BLM- or silica induced injury in AECs unlike uninjured control cells. This interaction has also been documented by others in different scenarios^27,29,44^. LC3B contains Cav1 binding motif (CBM) through which it interacts with Cav1^27,29^, and Cav1 scaffolding domain and intramembrane domain of Cav1 is pivotal for its interacting with LC3B^29^. A recent report from Bai X et al. suggests that the interaction of Cav1 scaffolding domain with LC3B will inhibit autophagy, which is reflected by decreased lipidation of LC3B^29^. This is consistent with our findings of CSP7 (a 7-mer fragment of Cav1 scaffolding domain peptide) treatment abrogating LC3BII levels in injured AECs. In addition, CSP7 treatment also decreases the interaction of p62 with Cav1 in injured AECs. This is not surprising as p62 harbors LC3 interaction region (LIR) through which it directly interacts with LC3B^5^ and their levels of expression are always concomitantly regulated. Moreover, studies showing the co-immunoprecipitation of p62 with Cav1/ubiquitin or with Cav1/LC3B/BID report the pivotal role of formation of this complex in regulation of selective autophagy and apoptosis^44,46,47^. In addition, Cav1 and LC3 interaction has also been shown to regulate extrinsic apoptosis via Fas in lung epithelial cells^27^. Based on the findings from the present study and our earlier works demonstrating CSP/CSP7 treatment preventing AECs apoptosis, we strongly believe that CSP7-mediated inhibition of autophagy dysregulation is paramount in alleviating apoptosis of AECs and thereby preventing PF development. In line with this, CSP7 mediated inhibition of p53, a master regulator of apoptosis was found to prevent dysregulation of autophagy in injured AECs. One of the limitations of the present study is that we did not perform long-term rescue/recovery study, it is therefore unclear whether mice with silica-induced PF without CSP7 treatment resolve spontaneously with time. Similarly, we did not use aged mice in our study that may respond differently to CSP7 treatment compared to younger mice. Further, the mice receiving CSP7 by IP injection may differ in their response from those administered via intravenous or subcutaneous or airway routes which is not explored in our study. Furthermore, dysregulated Cav1 expression has been reported in multiple human ailments, including cardiovascular and malignant diseases. Cav1-based therapies have shown promising results in several preclinical models. However, Cav1-based intervention is yet to be explored as a treatment for human diseases. Testing CSP7 for anti-fibrogenic activity using precision cut lung sections from patients with silicosis would increase human disease relevance. Formulation with excipients that could potentially increase the stability of CSP7 exhibiting low aqueous solubility are likely to enhance the suitability of CSP7 for lung delivery.

In summary, our data demonstrates that CSP7 prevents aberrant autophagy in injured AECs of multiple mouse models of PF through downregulation of the expression of p53 and by inhibiting the interaction of Cav1 with autophagy proteins LC3BII and p62. Thus, our study identifies CSP7 as a promising candidate for the treatment of dysregulated autophagy reported in various diseases including IPF and silicosis.

## Methods

### Mouse models of silica- and BLM-induced PF and CSP7 treatment

All experiments involving animals were performed in compliance with ARRIVE guidelines and in accordance with other relevant guidelines and regulations approved through protocols 630 and 701 by the Institutional Animal Care and Use Committee (IACUC) of The University of Texas Health Science Center at Tyler. Wild-type (WT) C57BL/6 J mice of six to 8 weeks old (weighing 20–25 g) were purchased from The Jackson Laboratory (Bar Harbor, ME) and were intratracheally administered with single dose (1X) of crystalline silica (MIN-U-SIL-5 @ 1.5 mg/100 μl) or saline (Sal) on day 1 and treated with or without CSP7 (1.5 mg/kg) by intraperitoneal (IP) injection from day 54 to day 60, (or) treated with 1X- or repeat dose (8X) of BLM (2 U/Kg) with or without CSP7 (1.5 mg/kg) as described previously^20^. To prepare isolated AECs for downstream assays, specific p53 conditional knockout (p53^cKO^) mice, homozygous surfactant protein C (SP-C)^cre^ p53 floxed (p53^fl/fl^) mice were administered with 100 ul tamoxifen/corn oil (75 mg/kg) by IP injection for 5 consecutive days. After a week, these mice were either administered with 1X-silica or 1X-BLM with or without CSP7 as described before. At the end of treatments, mice were euthanized, and lungs were harvested for preparation of homogenates, tissue sections for histopathologic evaluation or for isolation of AECs for ex vivo analysis.

### Treatment of human alveolar epithelial type II cells in vitro

Human AECs **(**hAECs) isolated from normal human lung tissue were purchased from Cell Biologics (Chicago, IL) and cultured as described elsewhere^17^ were treated with silica (100 µg/ml) for 72 h with or without CSP7 (20 μM) treatment for last 24- or 48 h, or with BLM (40 μg/ml) for 24 h with or without CSP7 (10 μM) treatment for last 4 or 8 h. For autophagy flux analysis, 30 µg/ml of chloroquine (CQ) was used as positive control.

### Sircol assay and Hydroxyproline quantification

The amount of soluble collagen in the mouse whole lung tissues was determined using a Sircol Collagen Assay Kit (Biocolor Ltd, Carrickfergus, UK) and the hydroxyproline content was quantified using previously described methods^17,20,33^.

### Trichrome staining and immunohistochemistry

For trichrome and immunohistochemistry (IHC) analysis, mouse lungs were harvested and fixed using Excell Plus solution (American MasterTech Scientific, Inc., Lodi, CA). The paraffin embedded lung tissue blocks were then cut into 5 μm thick sections, deparaffinized, rehydrated and stained for collagen and other matrix protein using Masson’s trichrome, or antigen retrieval was performed and stained for LC3B (1:100, abcam, USA) and p62 (1:100, Millipore sigma, USA) proteins as described^17^.

### Western blotting

Whole lung homogenates and AEC lysates were separated on SDS-PAGE and electroblotted onto a nitrocellulose membrane. After blocking with 1% bovine serum albumin (BSA) for 1 h, membranes were incubated with primary antibodies at respective dilutions at 4^o^ C overnight, followed by washing and incubation with donkey anti-rabbit/Mouse/Goat horseradish peroxidase conjugated secondary antibody at 1:2000 dilution for 1 h at room temperature in 5% milk buffer. After washing, the protein was visualized using ECL detection method^16–18,20^. Some blots were cropped before incubation with primary antibodies. List of antibodies and dilution used are provided in the online supplement.

### RNA isolation and quantitative real time PCR (qPCR)

Total RNA extracted from lung homogenates was reverse transcribed and subjected to qPCR for Col1, α-SMA, LC3B, p62 and ATG5 transcripts^17,20^. qPCR primers were purchased from Bio-Rad (Hercules, CA).

### Statistical analysis

GraphPad Prism software version 9 was used for data analysis. Statistical significance between multiple groups was compared using one-way ANOVA with Turkey’s multiple comparison test. *P* < 0.05 was considered significant.

## Supplementary Information

Supplementary Information 1.

Supplementary Information 2.

## Data Availability

Data used in this Study are available from the corresponding author upon request.
