# Supplementary figures and images for: Caveolin-1 scaffolding domain peptide abrogates autophagy dysregulation in pulmonary fibrosis
Source: Sci Rep. 2022 Jun 30;12:11086. doi: 10.1038/s41598-022-14832-4 (PMC9246916; doi:10.1038/s41598-022-14832-4)

Fig 1E

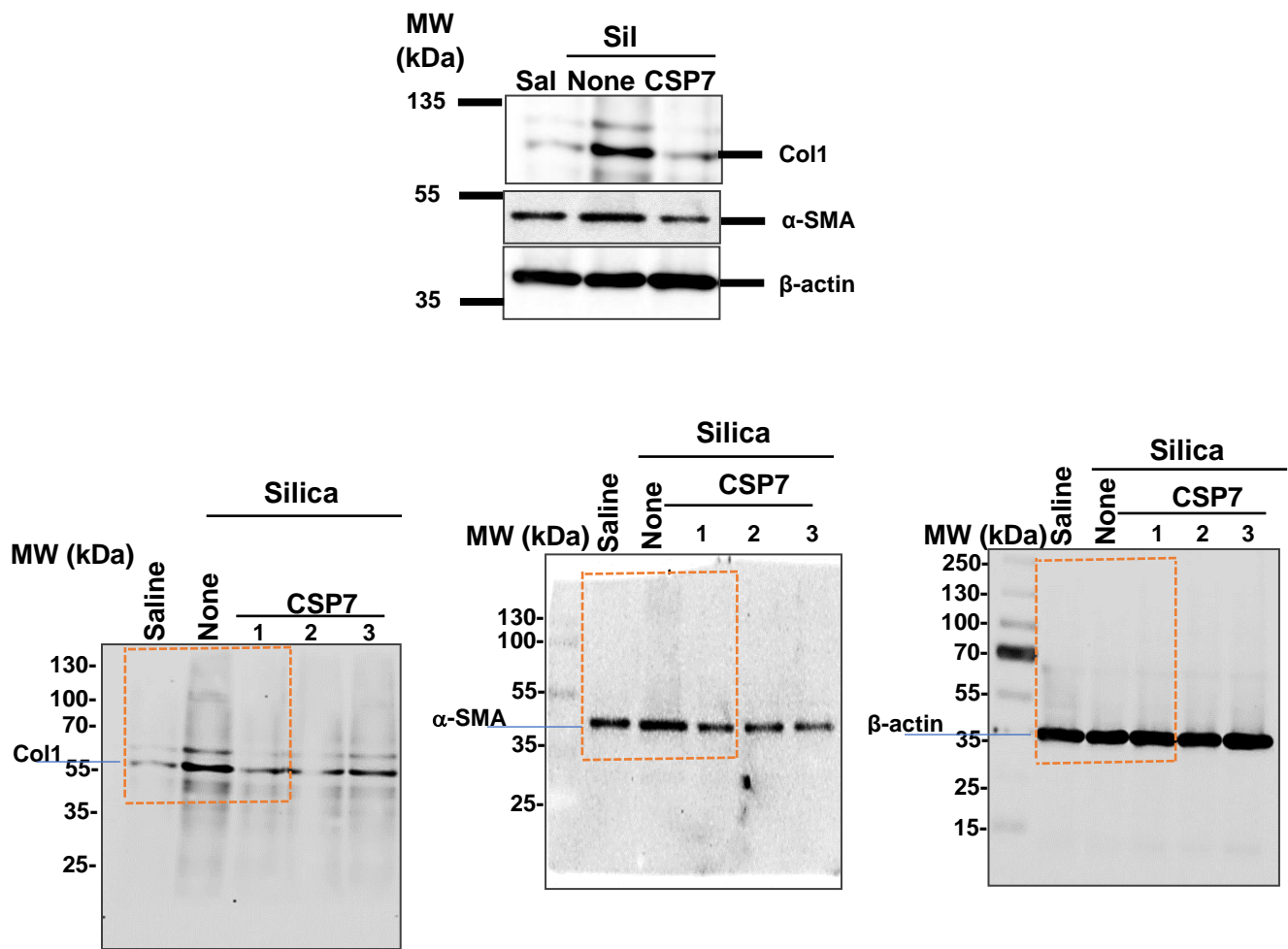

Fig 2A

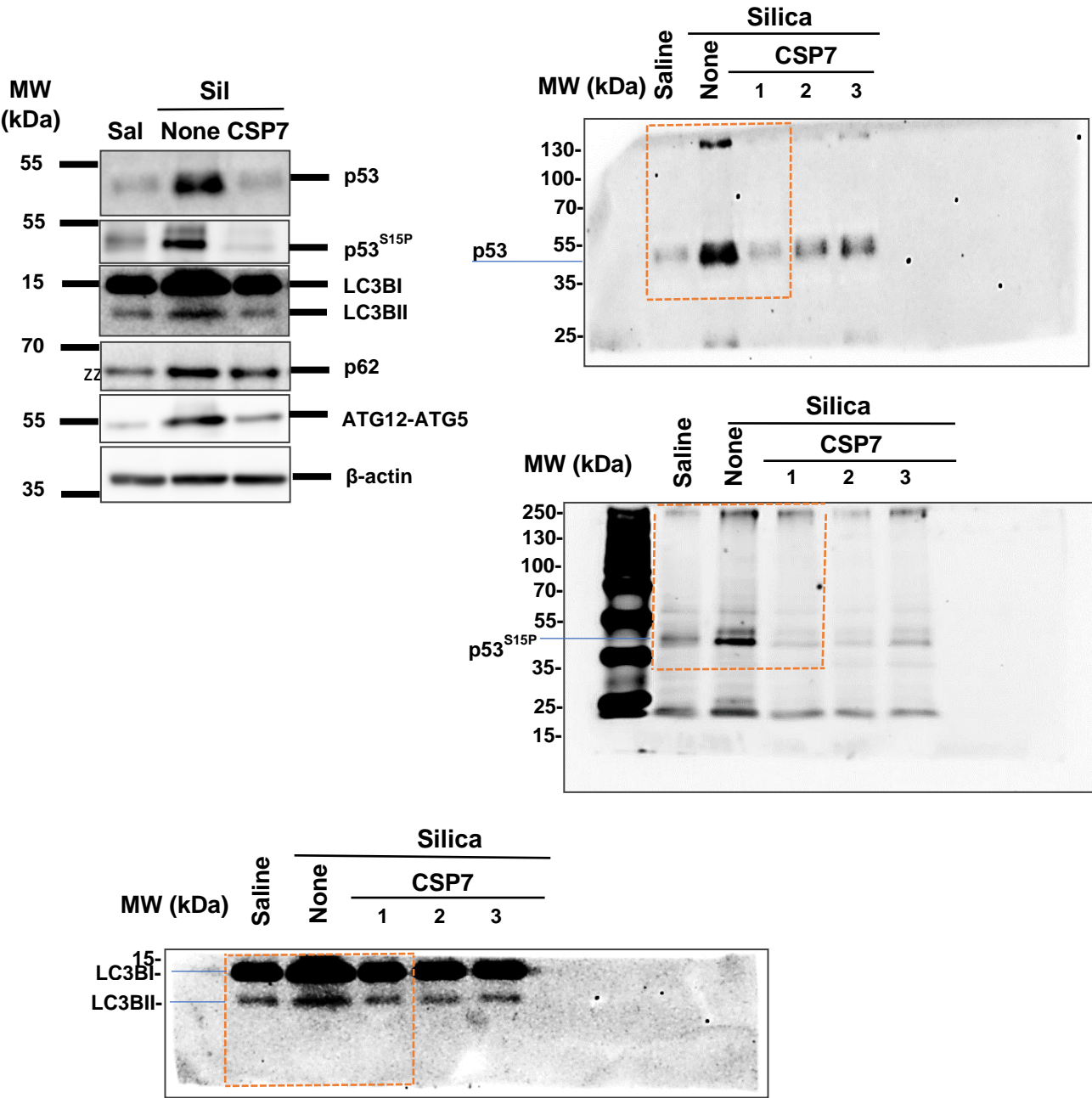

Fig 2A

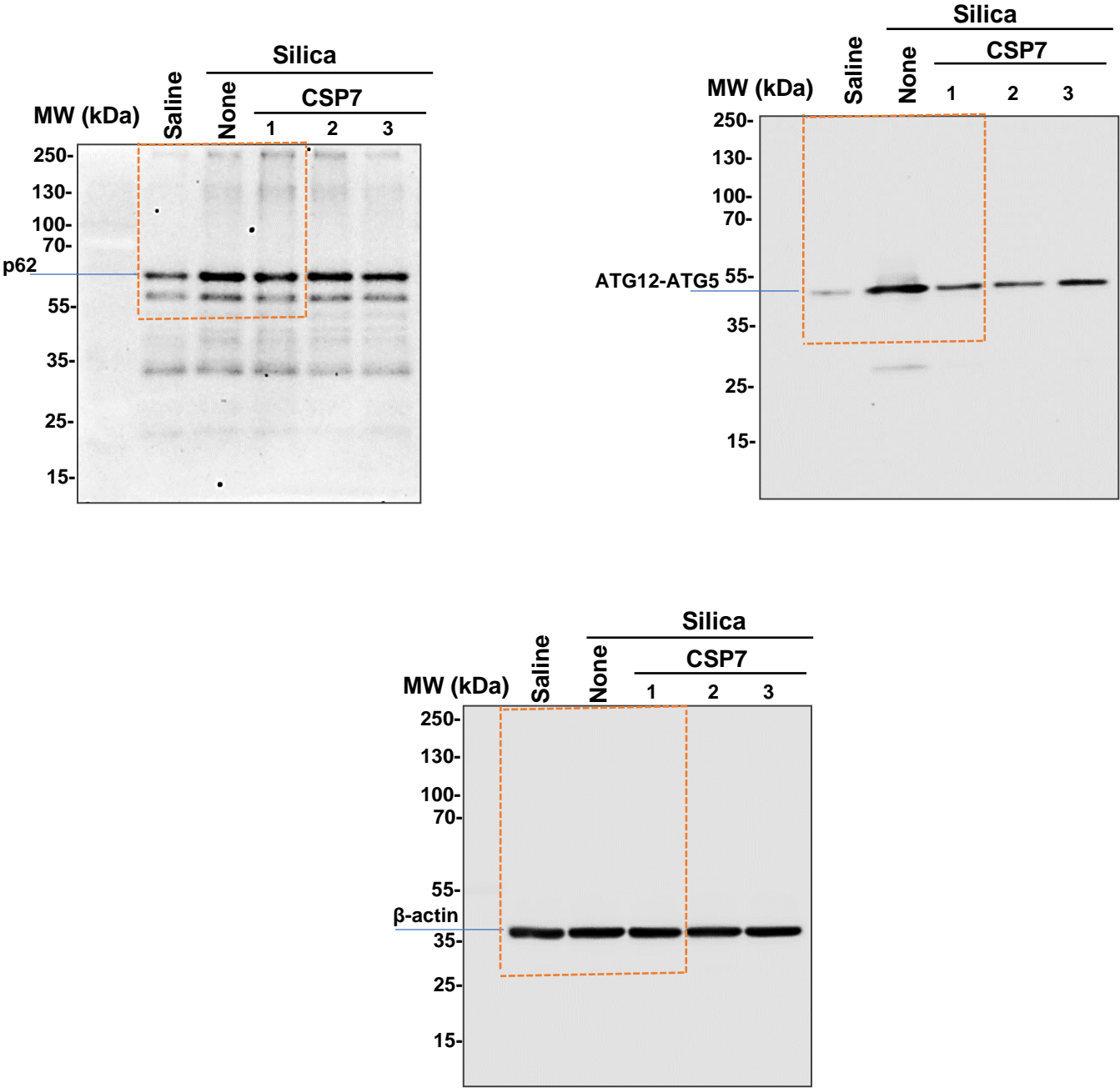

Fig 2E

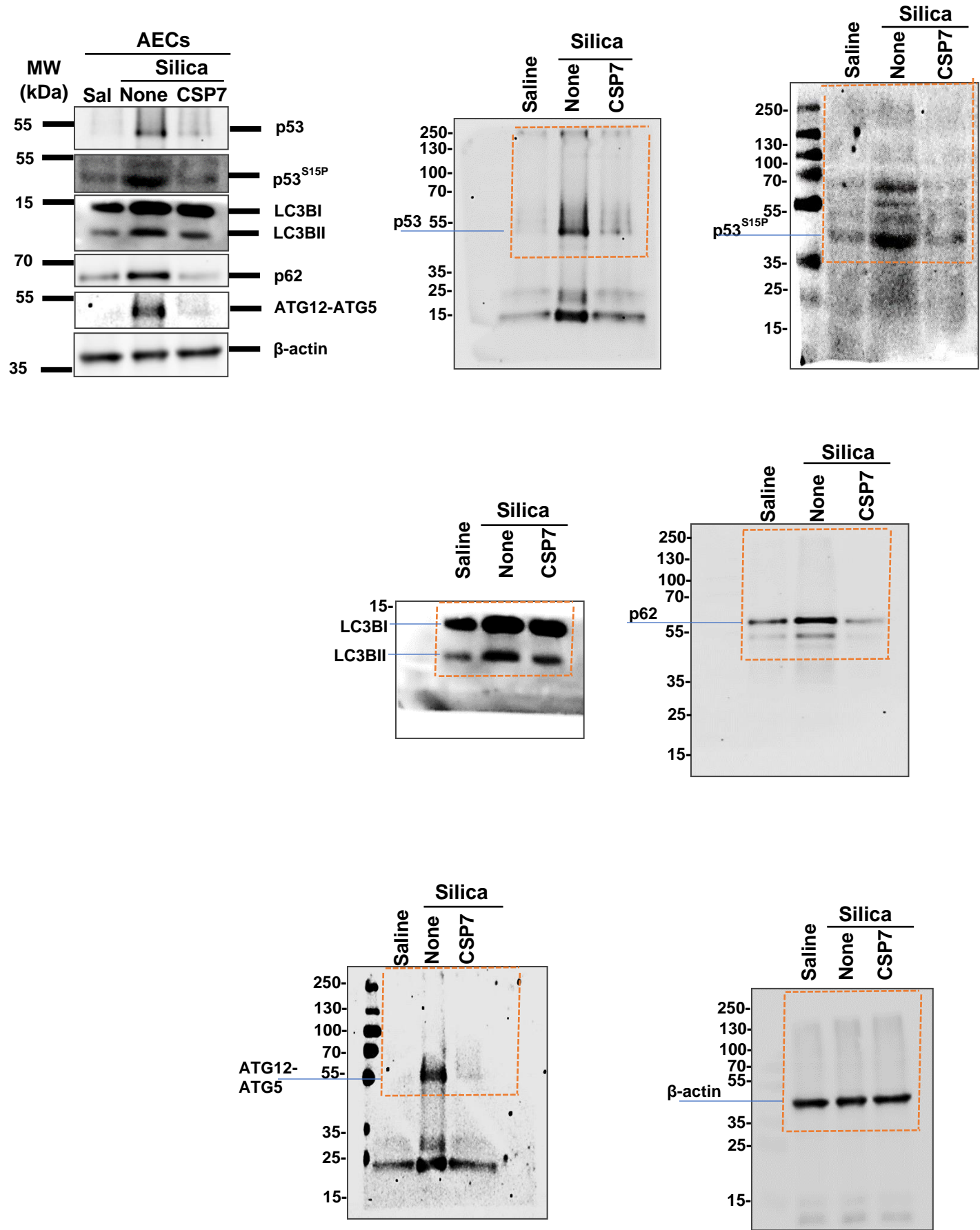

Fig 3A

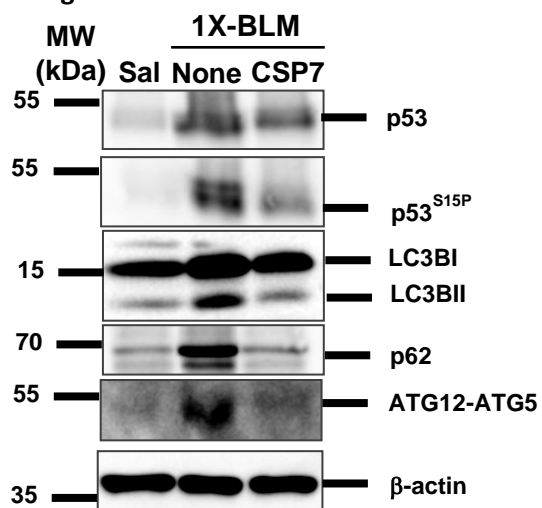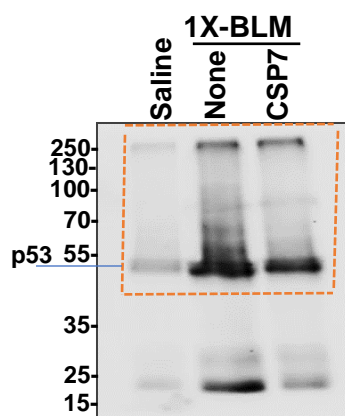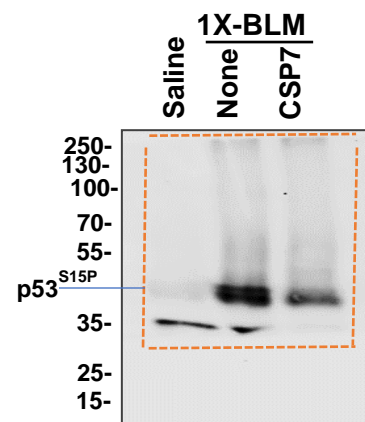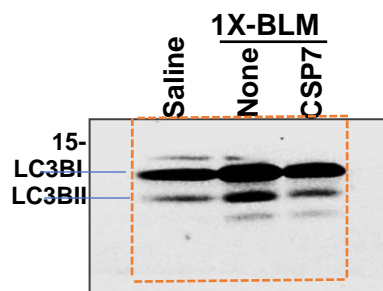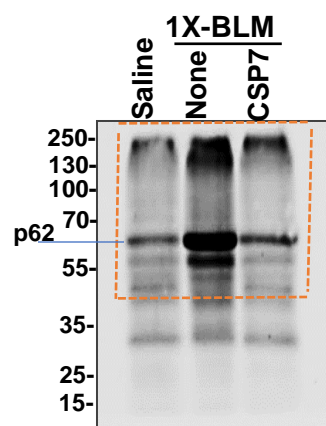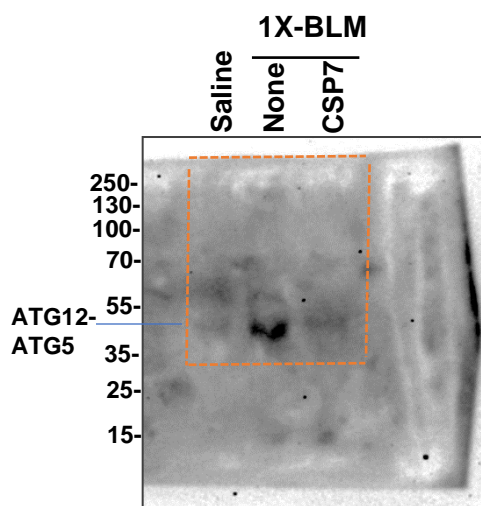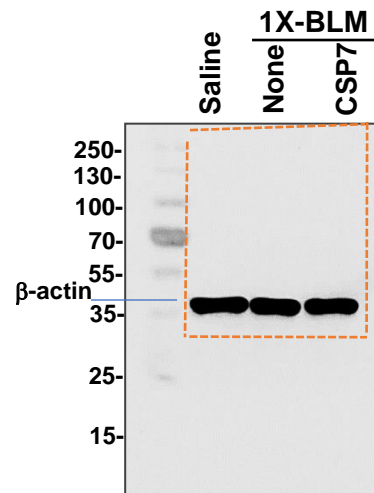

Fig 3E

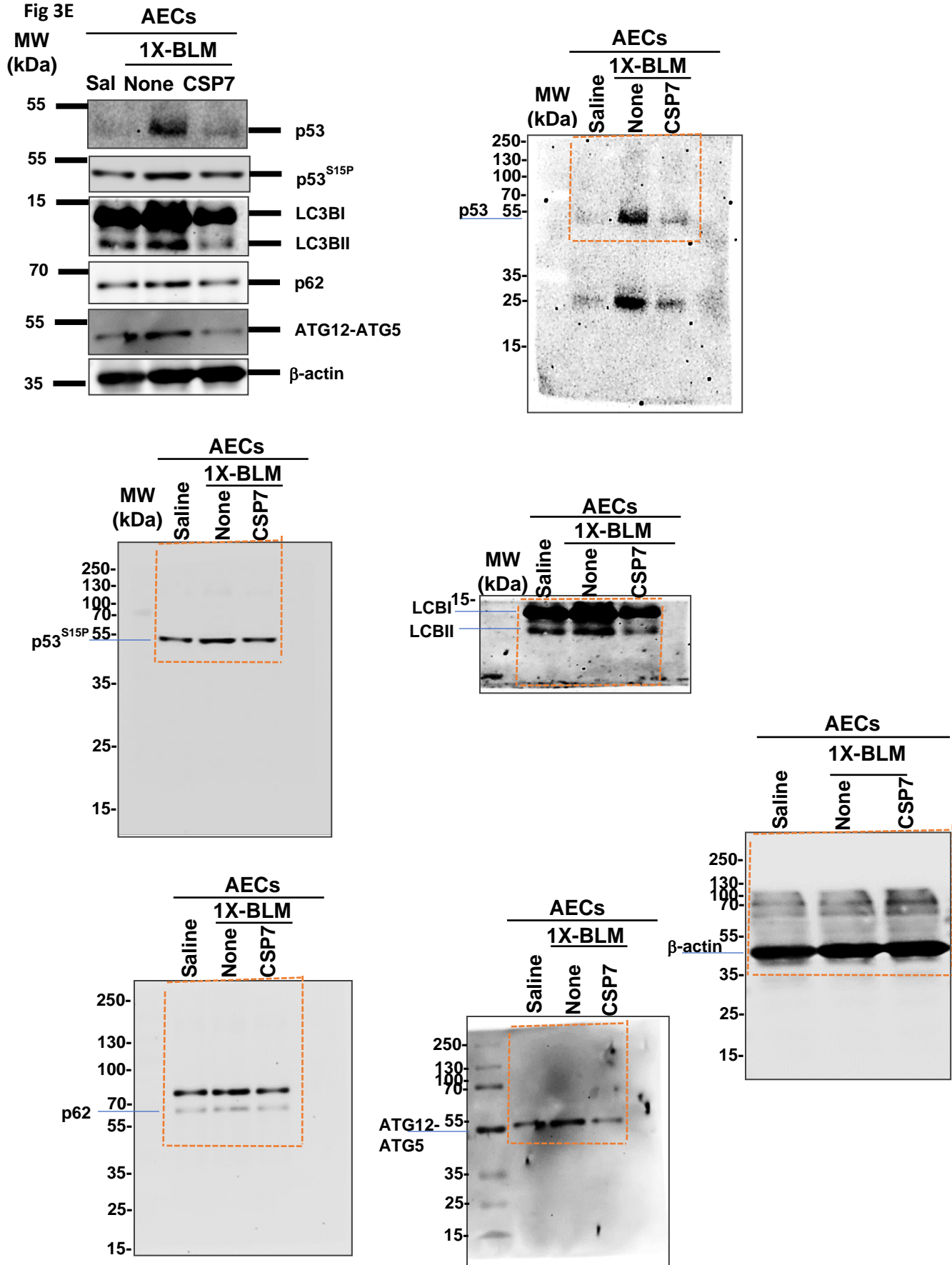

Fig 4A

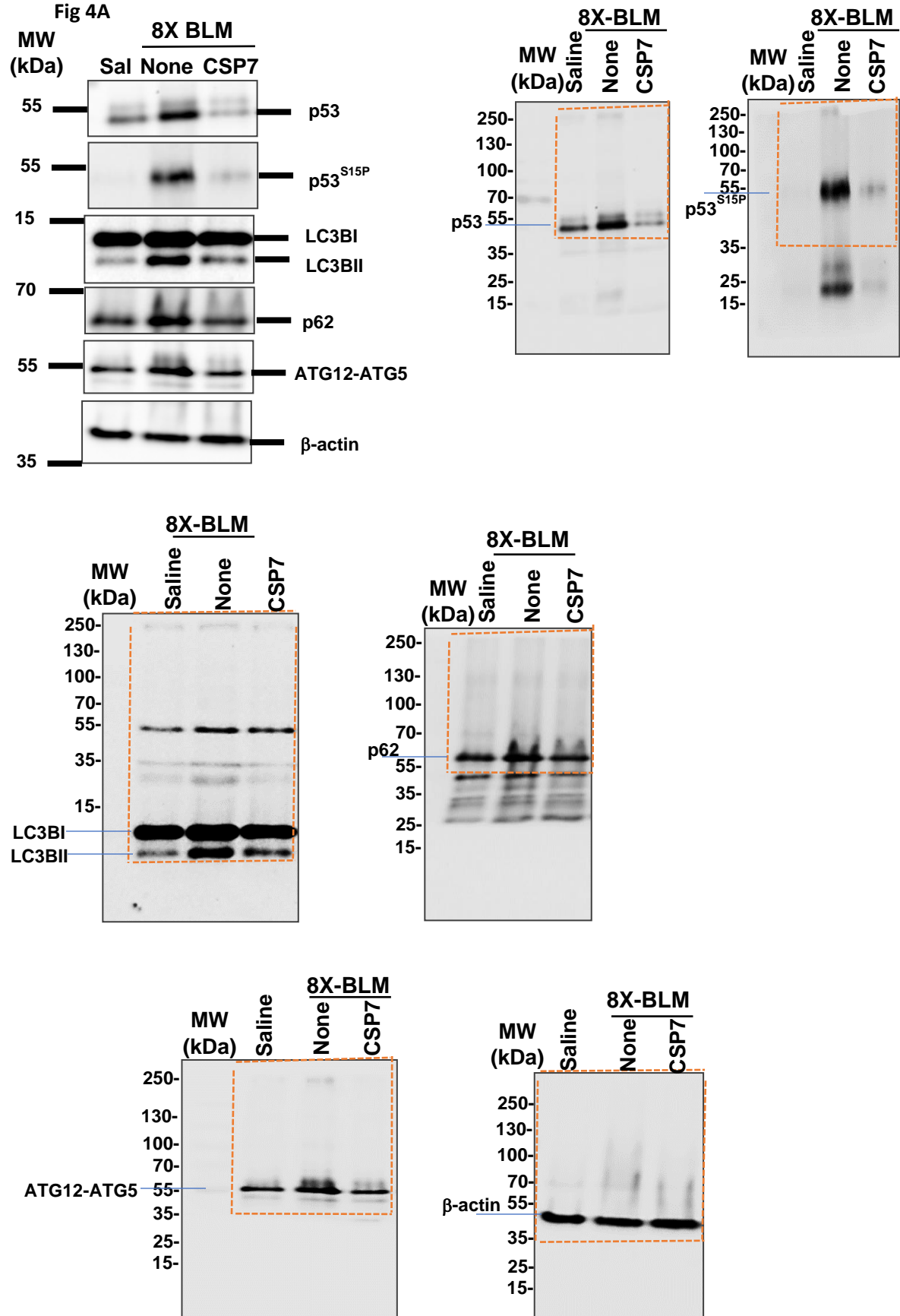

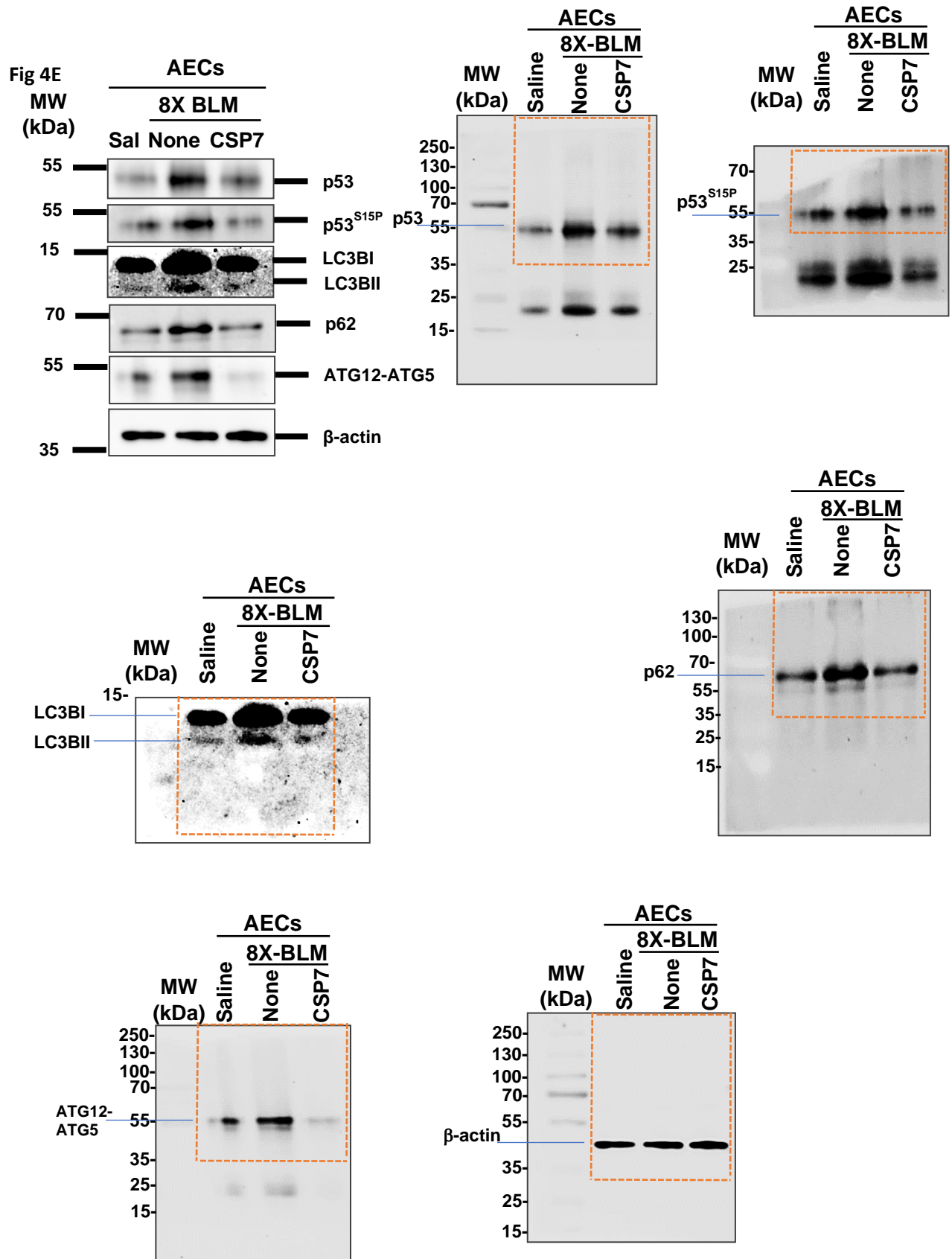

Fig 5A

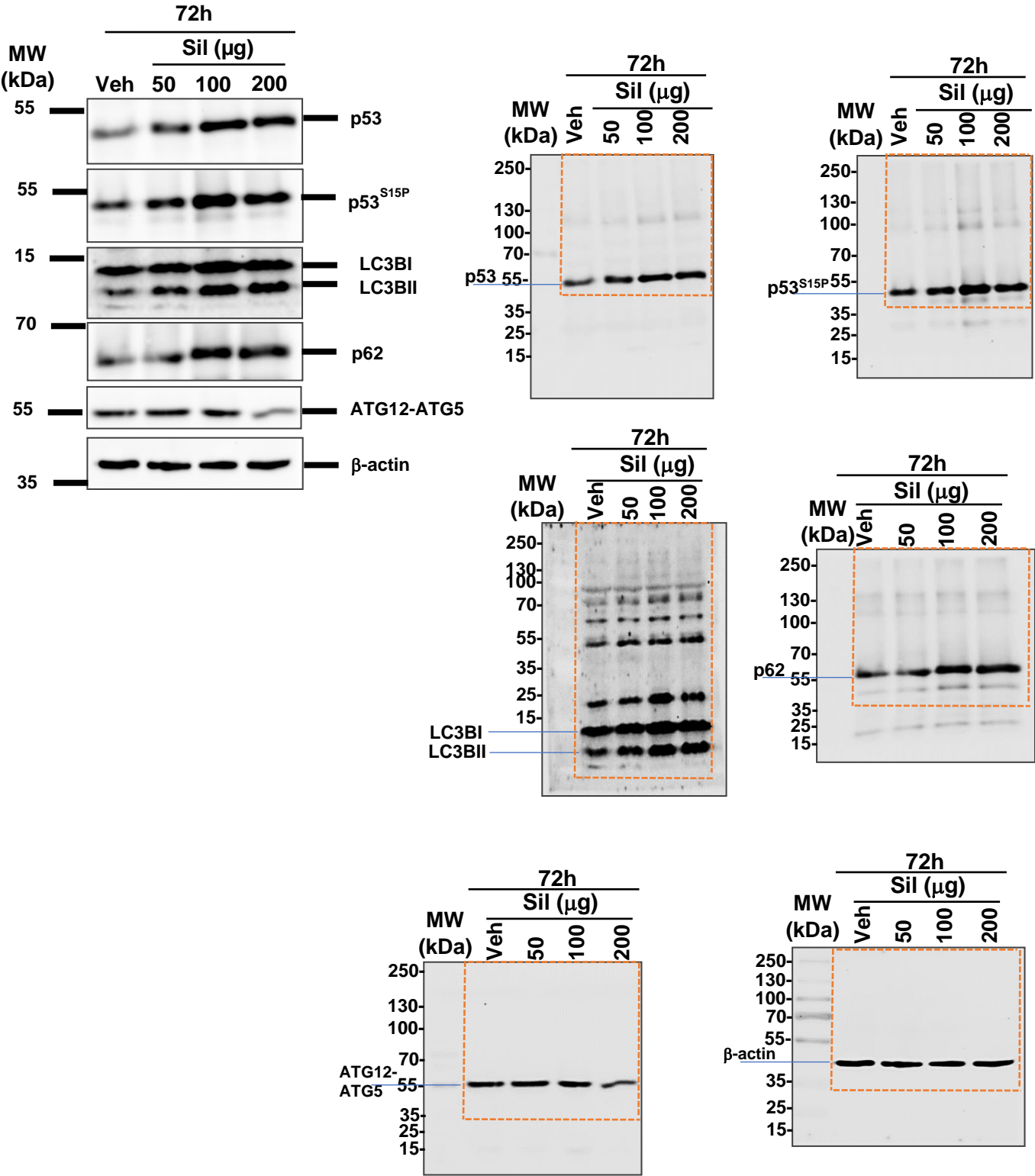

Fig 5B

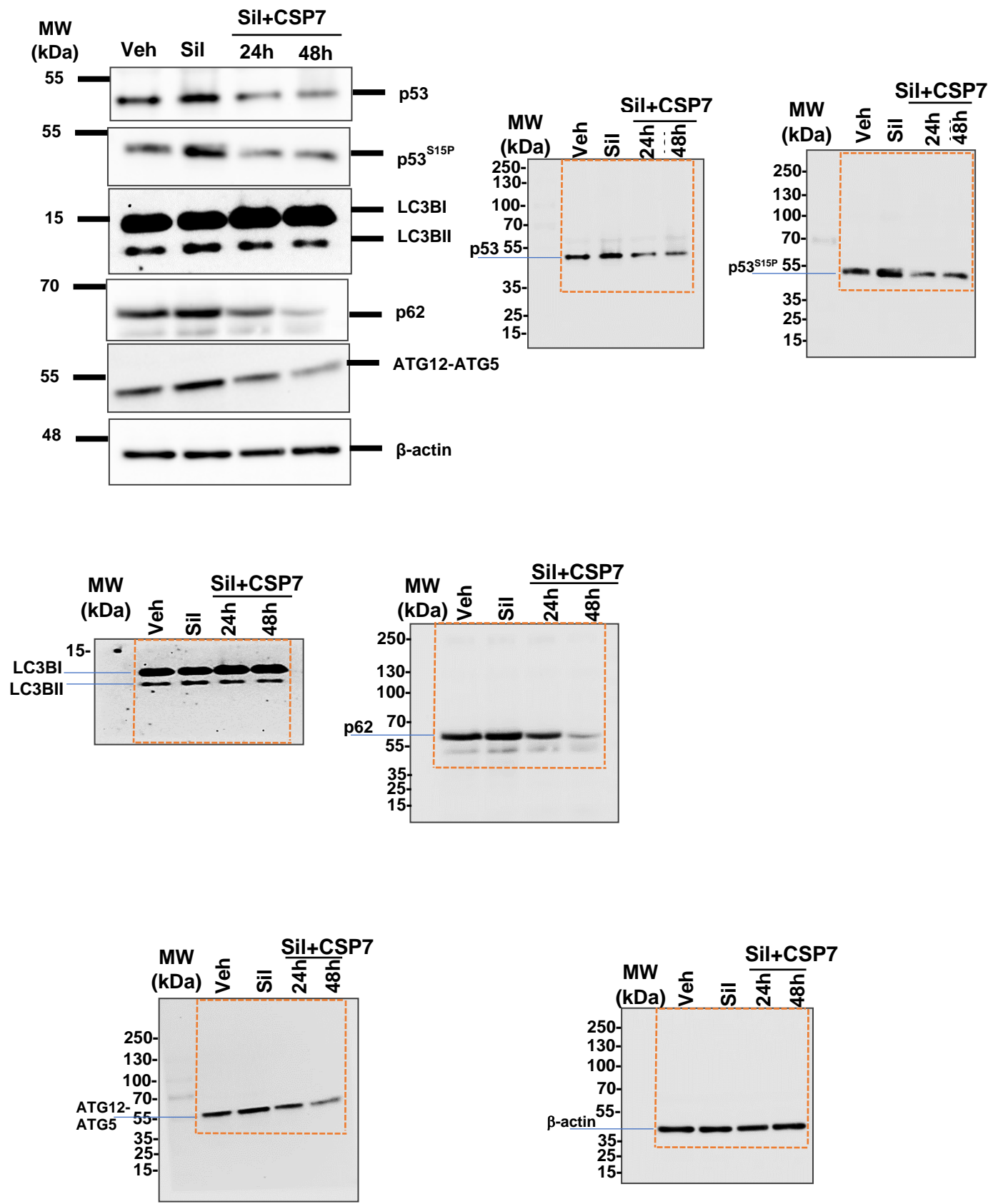

Fig 5C

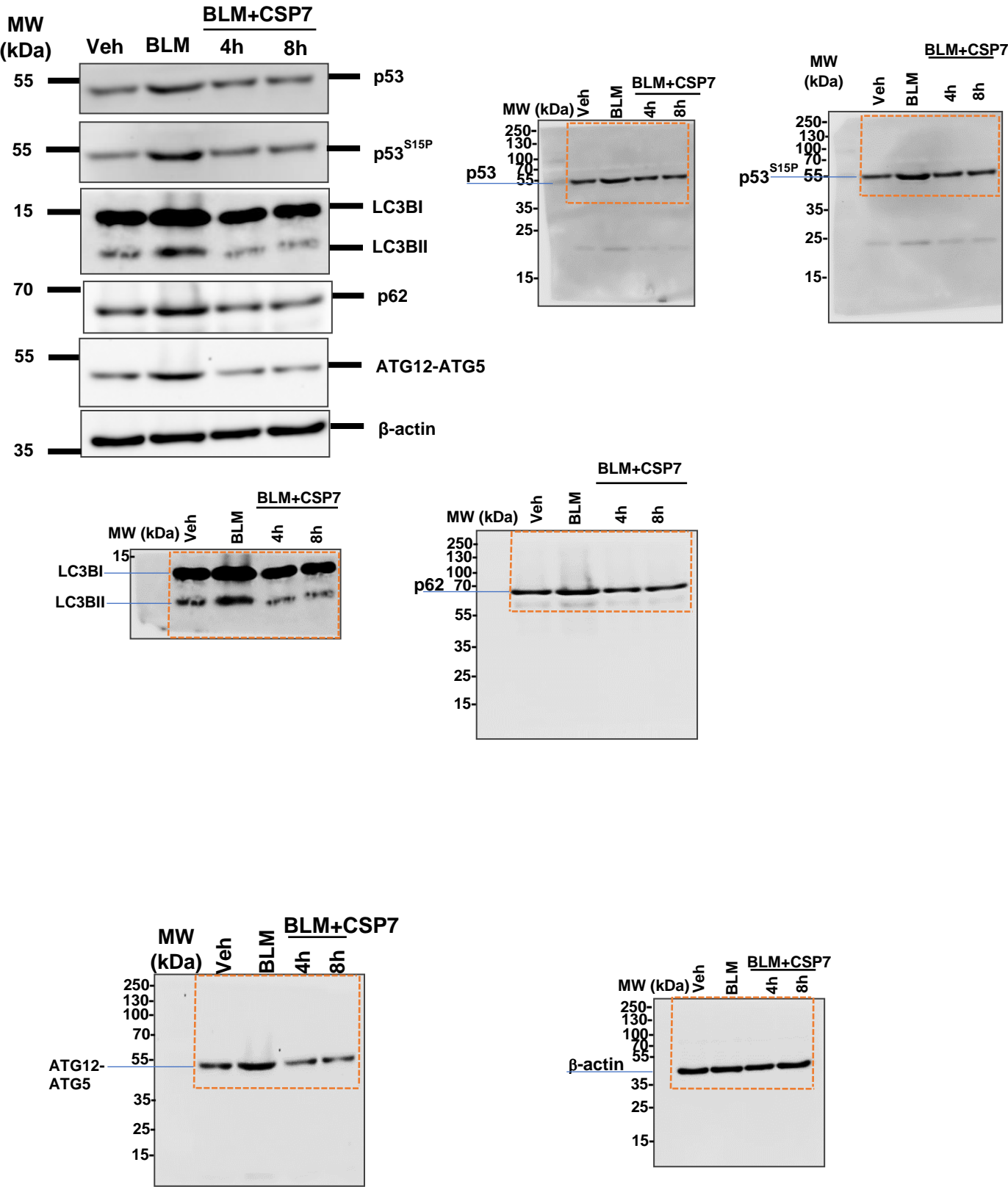

Fig 5D

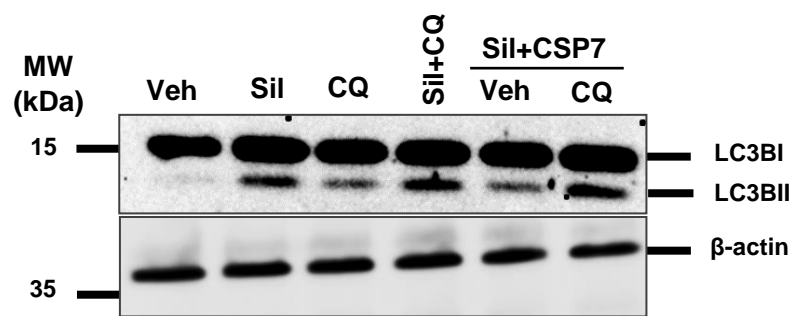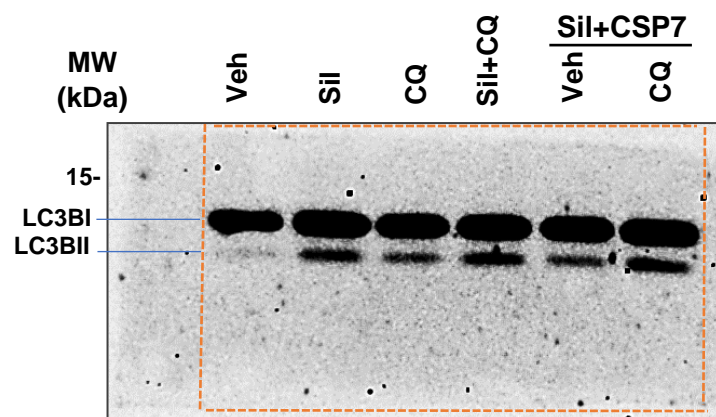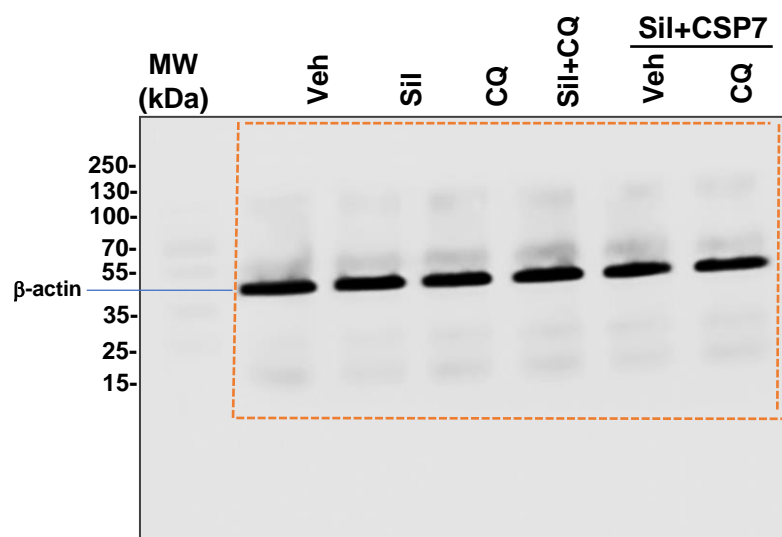

Fig 5E

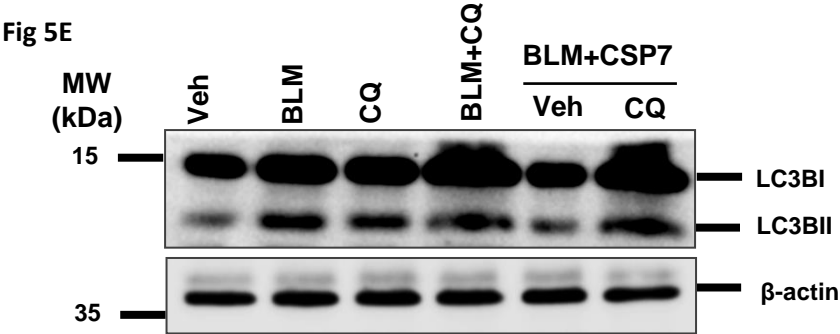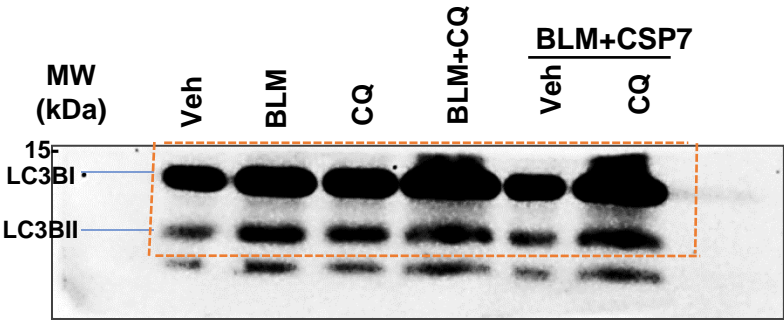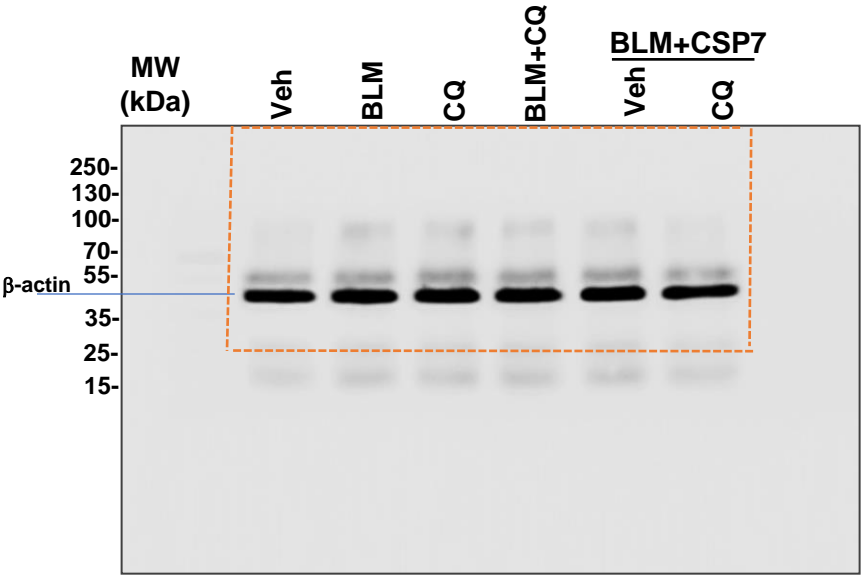

Fig 5F

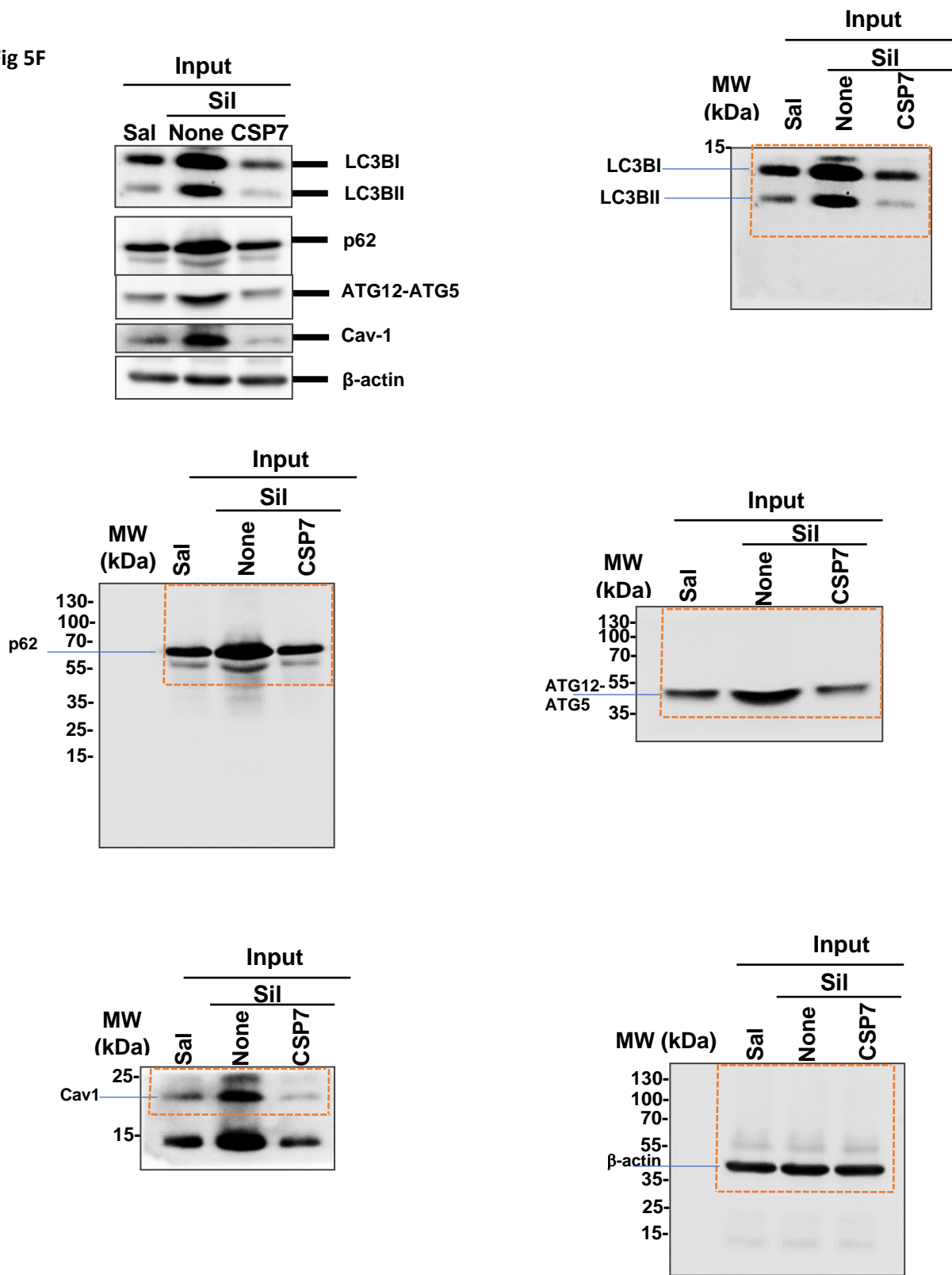

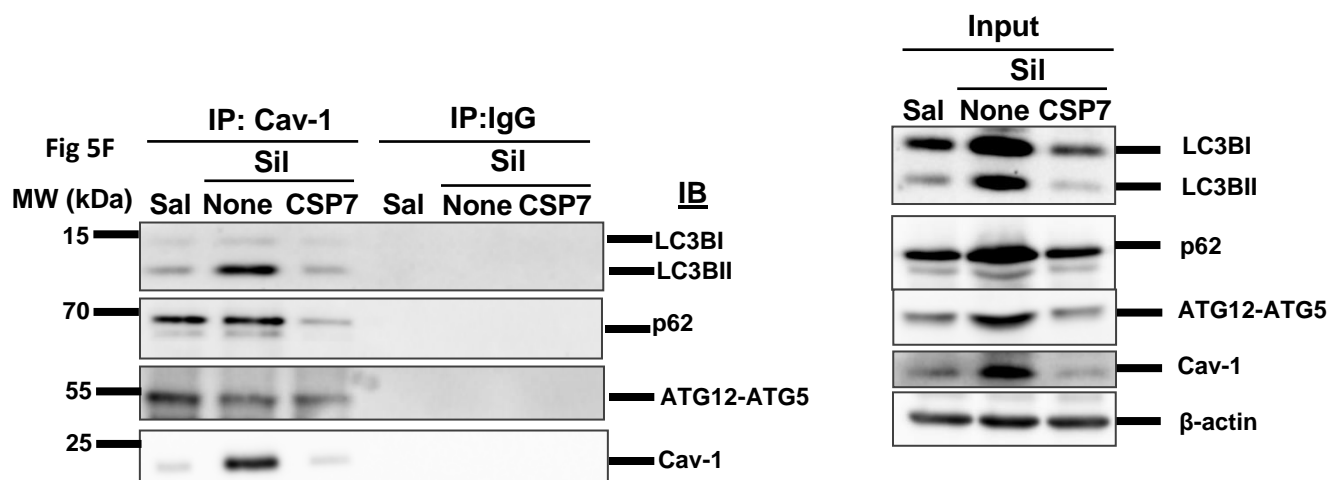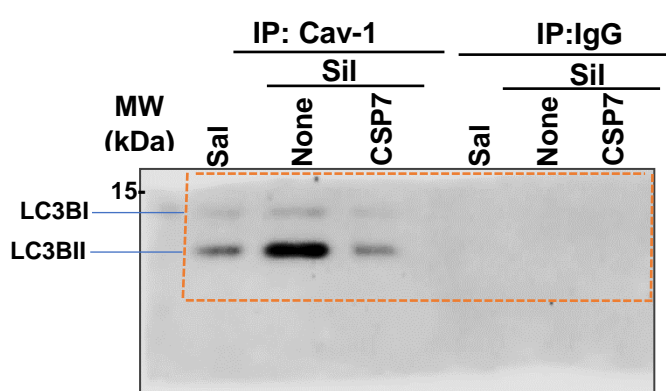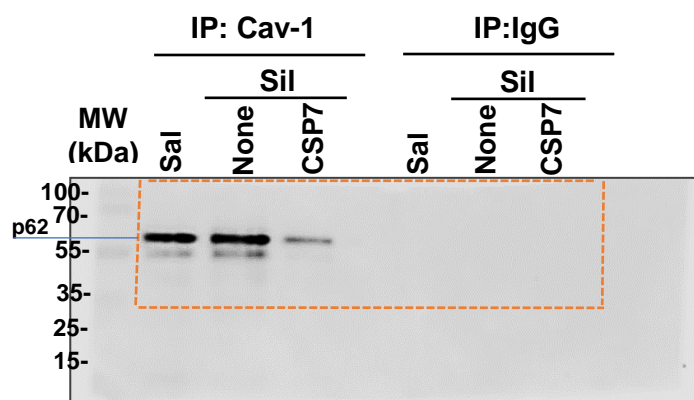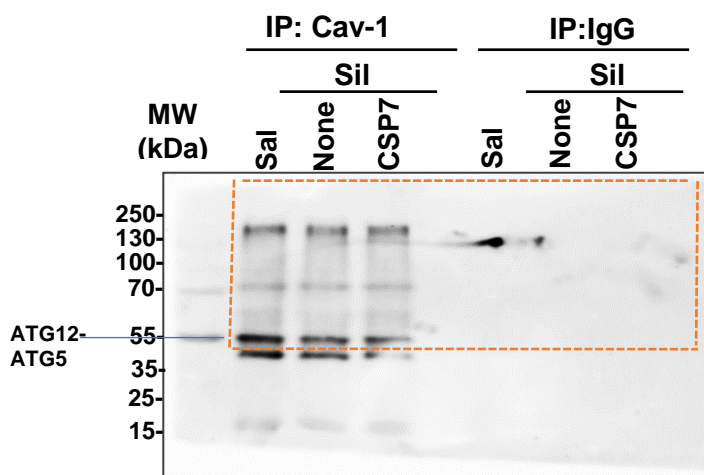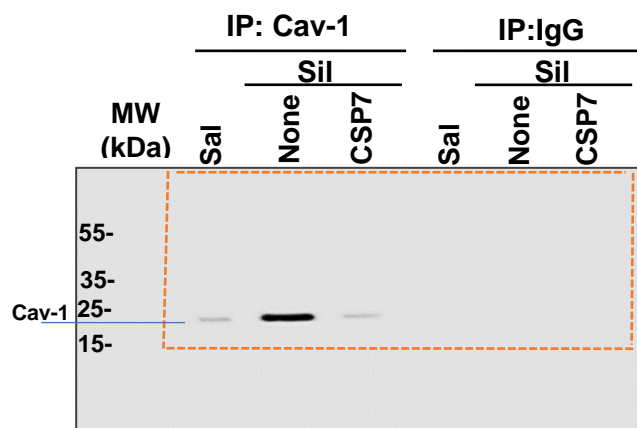

Fig 5G

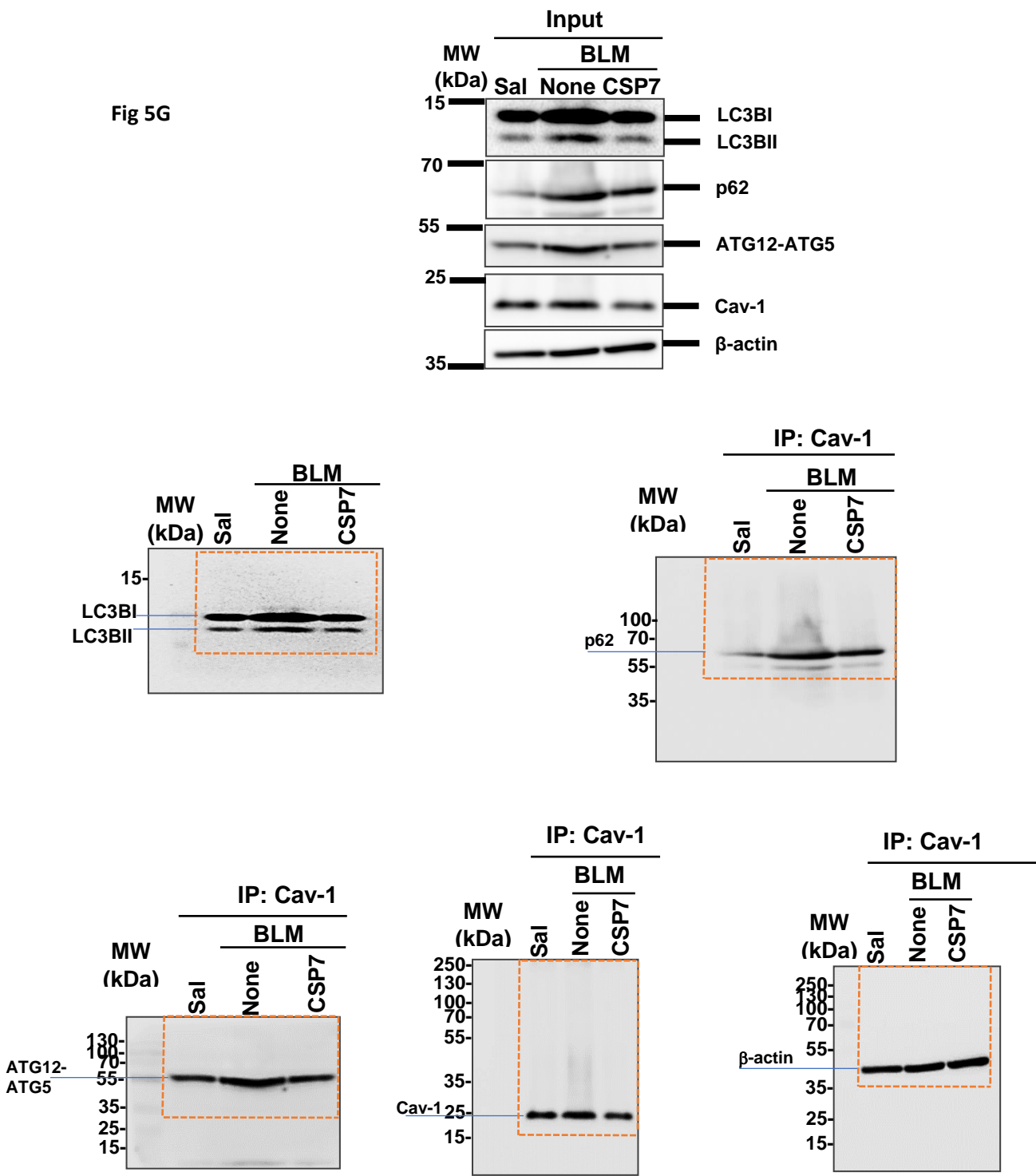

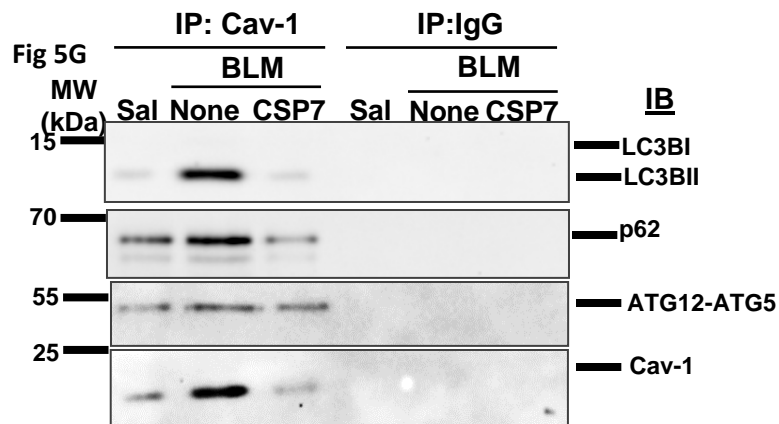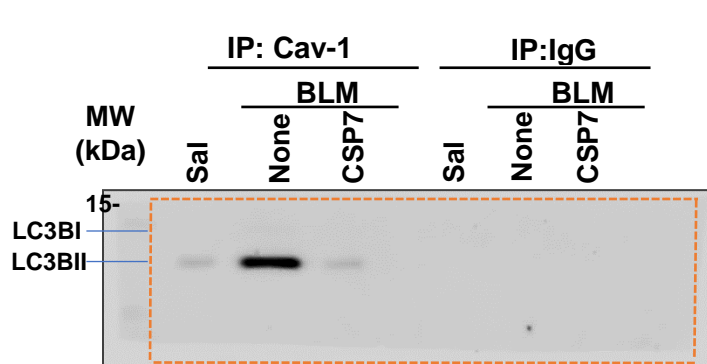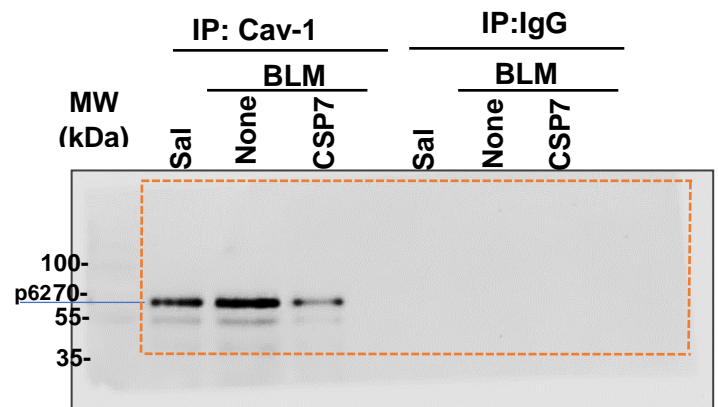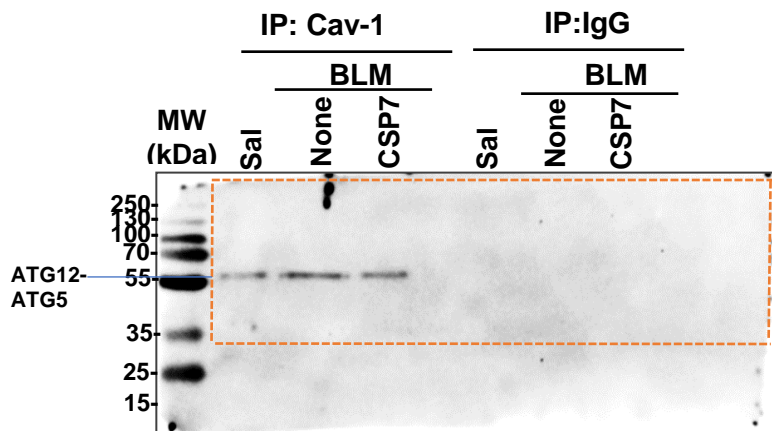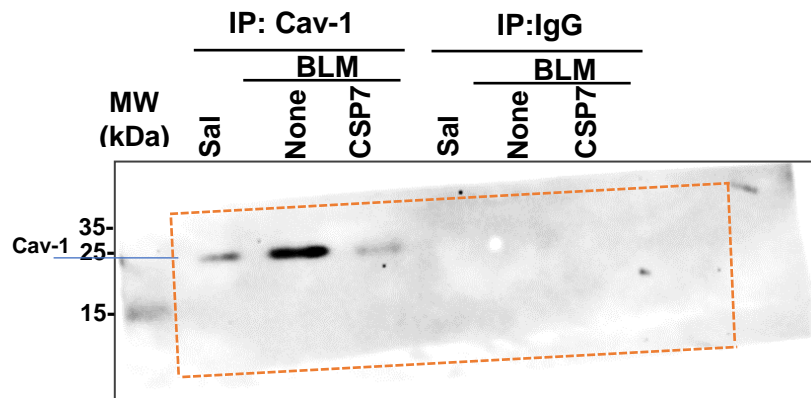

Fig 6A

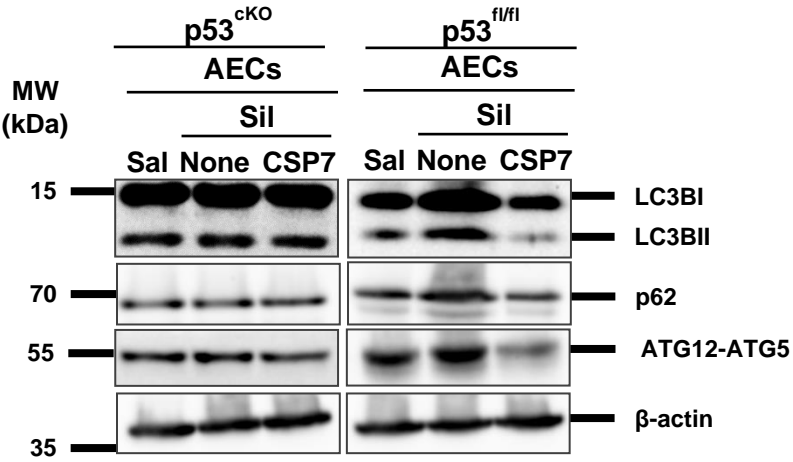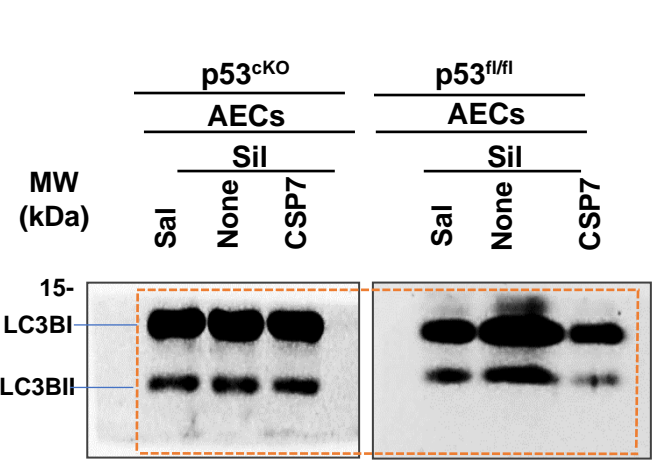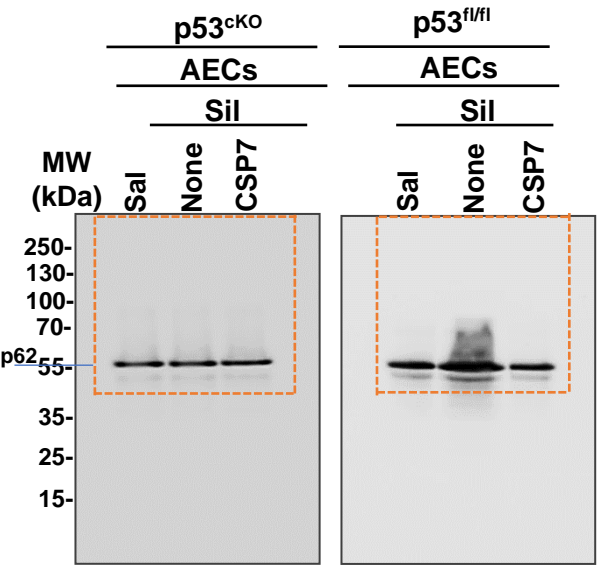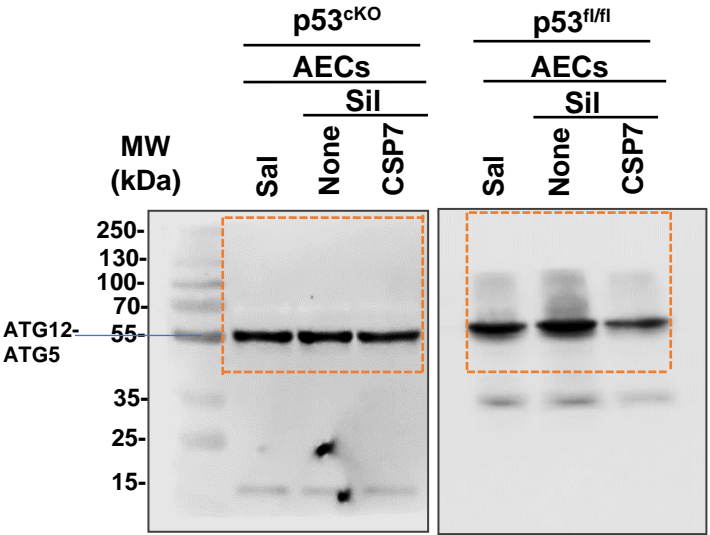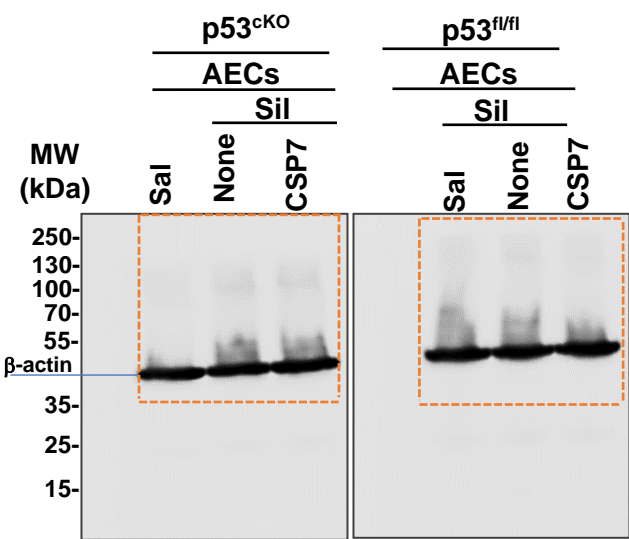

Fig 6B

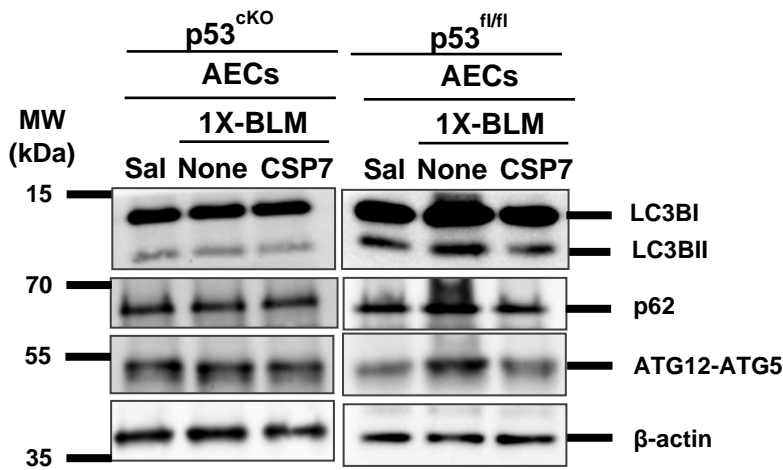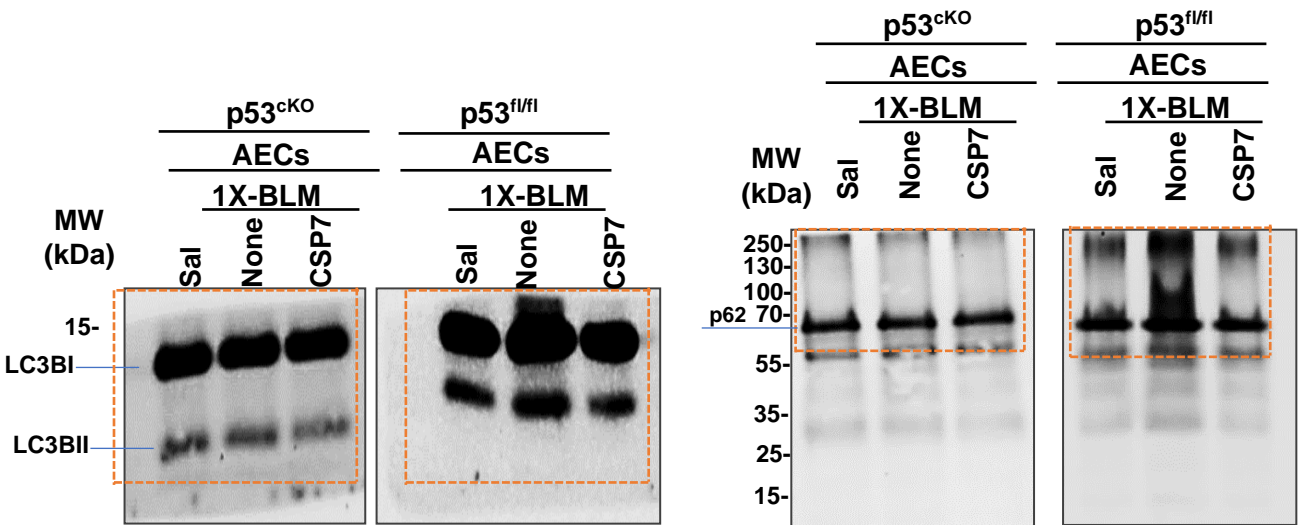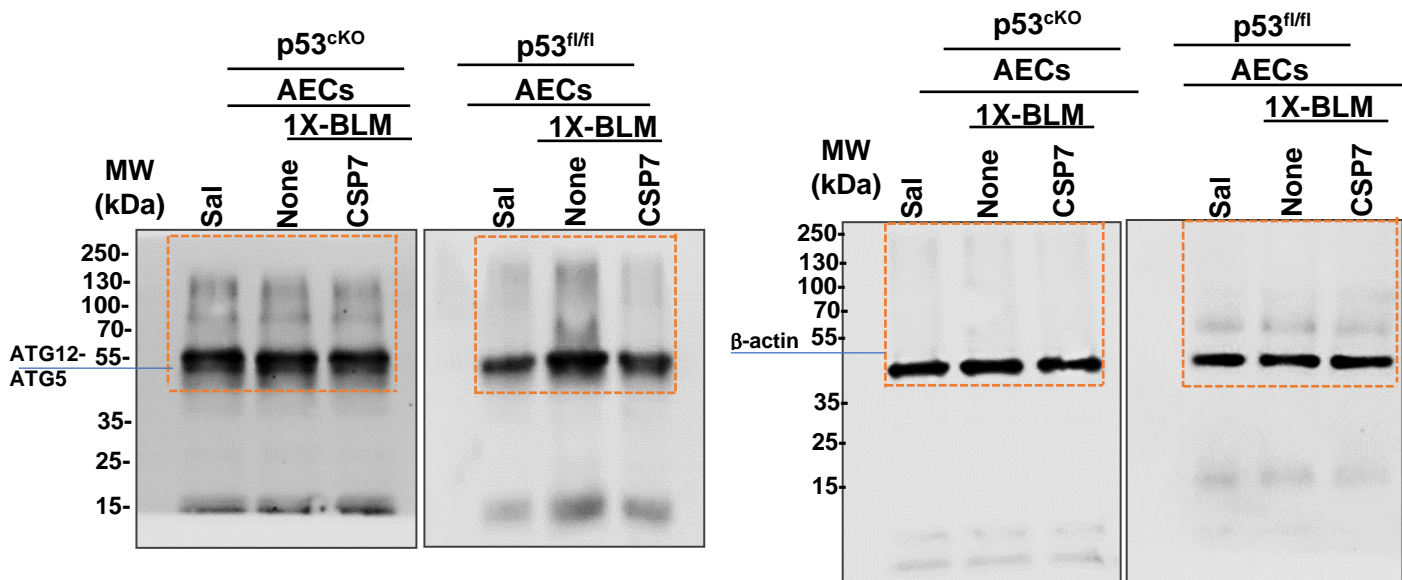

Fig S2

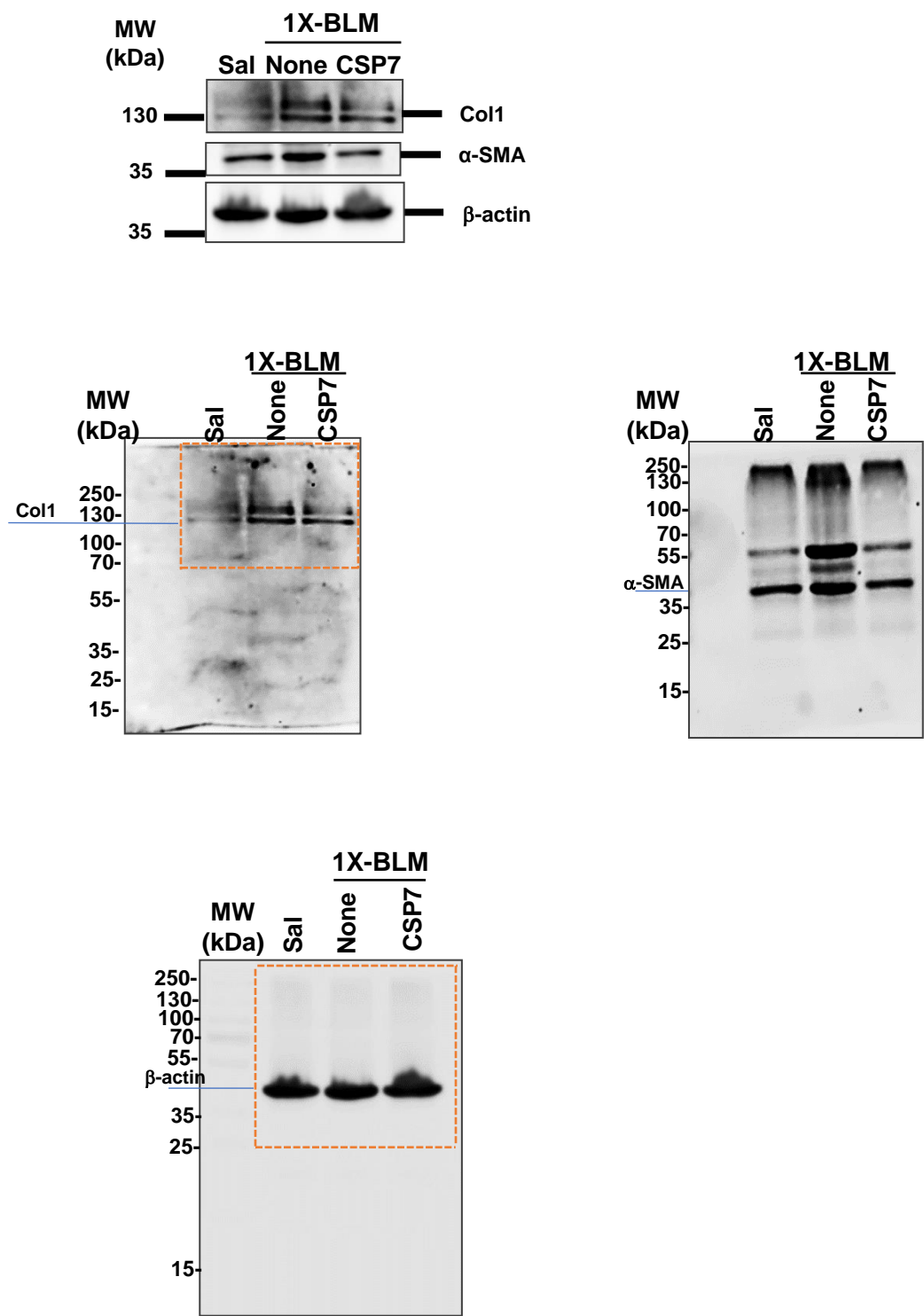

Fig S3

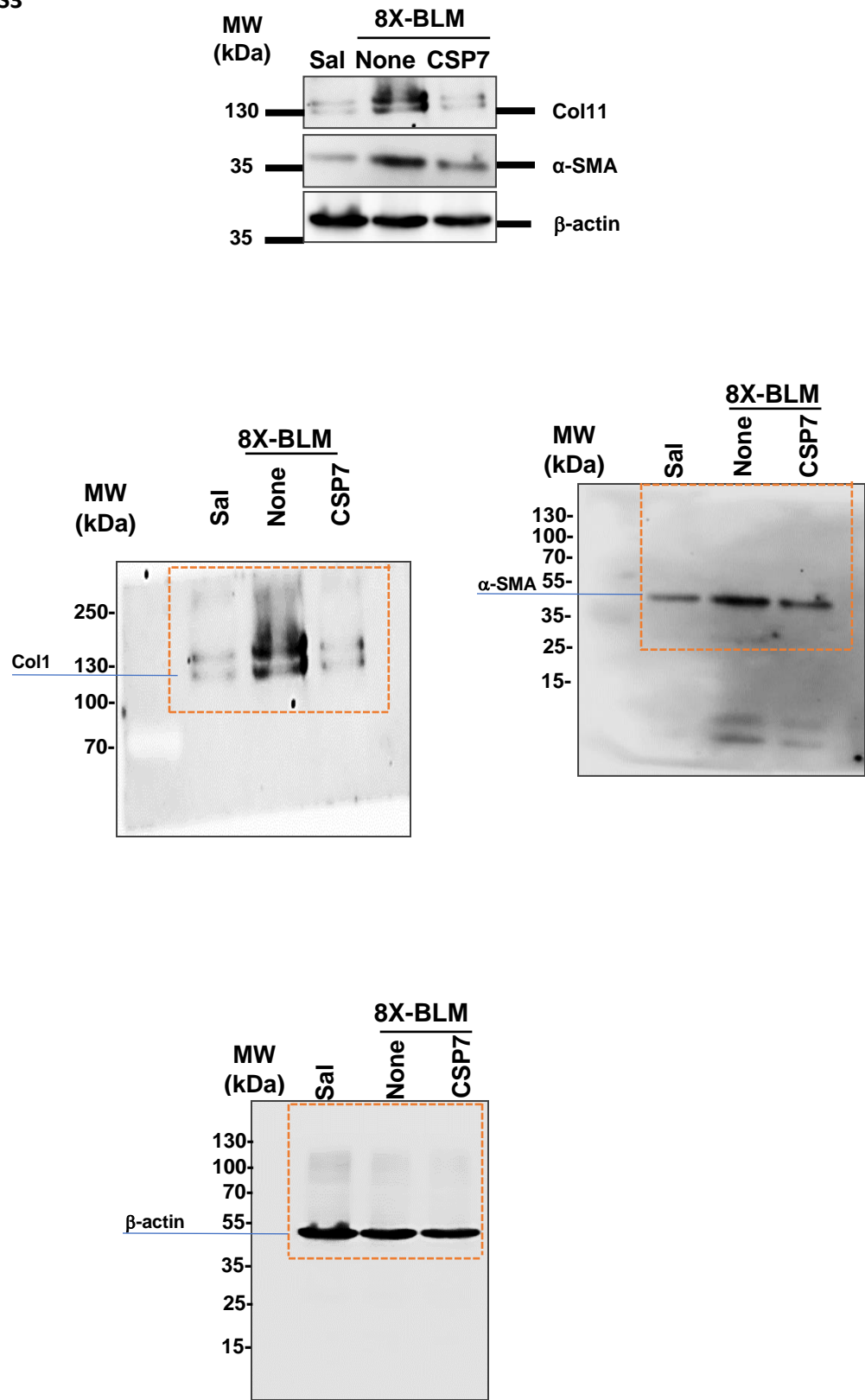

Supplement: Supplementary file 1 — Supplementary Information 1. [file 41598_2022_14832_MOESM1_ESM.pdf]
