# Supplementary material for: Caveolin-1 scaffolding domain peptide abrogates autophagy dysregulation in pulmonary fibrosis
Source: Sci Rep. 2022 Jun 30;12:11086. doi: 10.1038/s41598-022-14832-4 (PMC9246916; doi:10.1038/s41598-022-14832-4)
Supplement: Supplementary file 2 — Supplementary Information 2. [file 41598_2022_14832_MOESM2_ESM.docx]

**Caveolin-1 scaffolding domain peptide abrogates autophagy dysregulation in pulmonary fibrosis**

Shalini Venkatesan^1^, Liang Fan^1^, Hua Tang, Nagarjun V. Konduru and Sreerama Shetty^1*^

^1^Texas Lung Injury Institute, Department of Medicine, University of Texas Health Science Center at Tyler, 11937 US Highway 271, Tyler, TX 75708.

*Correspondence: [sreerama.shetty@uthct.edu](mailto:sreerama.shetty@uthct.edu)

Phone: 903-877-7668

## **Supplemental Materials**


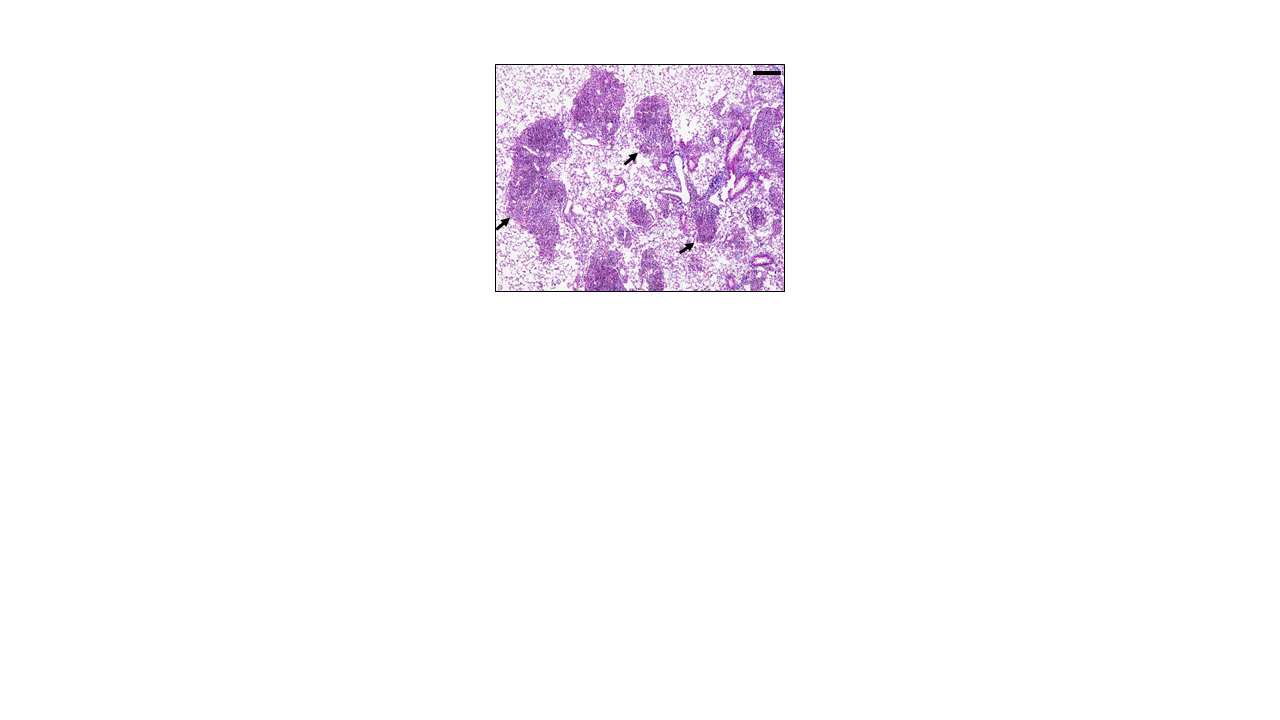


**Figure S1. Establishment of 1X-Silica-induced PF in WT mice.** Representative immunohistochemistry image (n = 5) showing fibrotic nodules (arrows) formed in murine model of 1X-silica-induced PF. Scale bar: 100 μm.


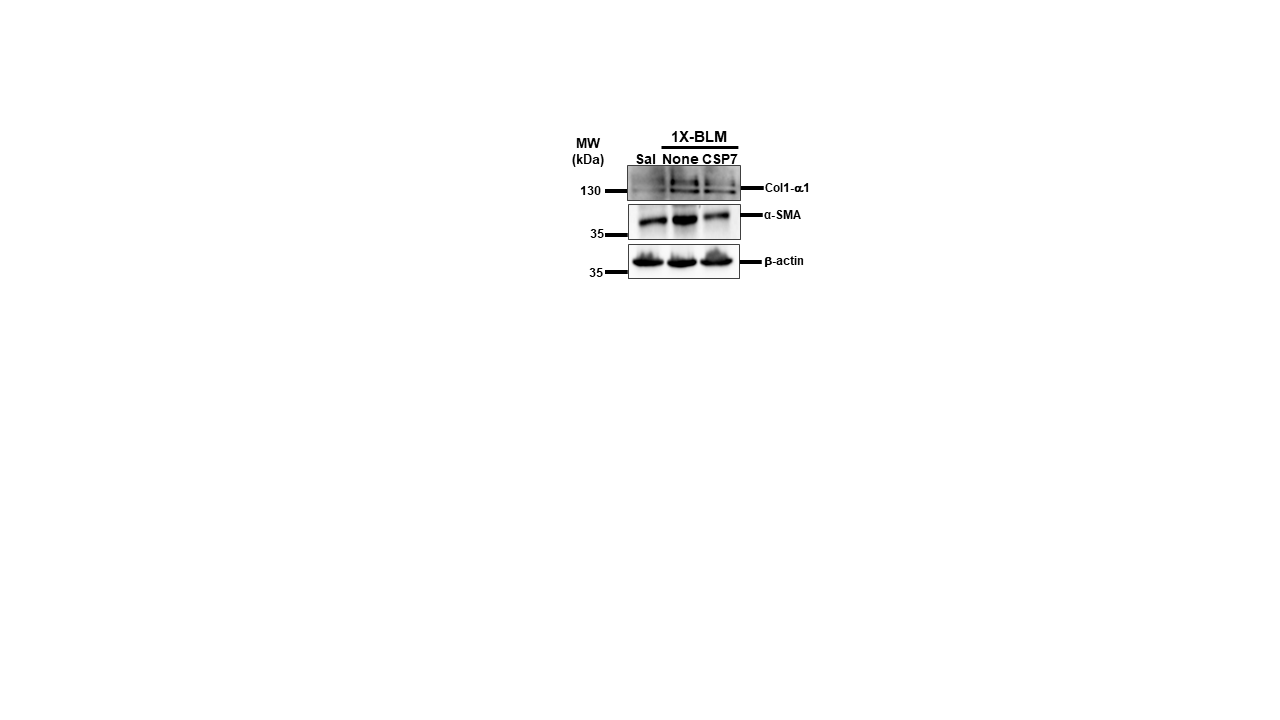
**Figure S2. Development of 1X-BLM-induced PF in WT mice.** Representative Western blot images showing the expression of Col1 and α-SMA in lung homogenates of WT mice exposed to saline or 1X-BLM or 1X-BLM+CSP7. MW: Molecular weight. kDa: Kilodalton. The experiment was repeated at least 2 times with similar results.


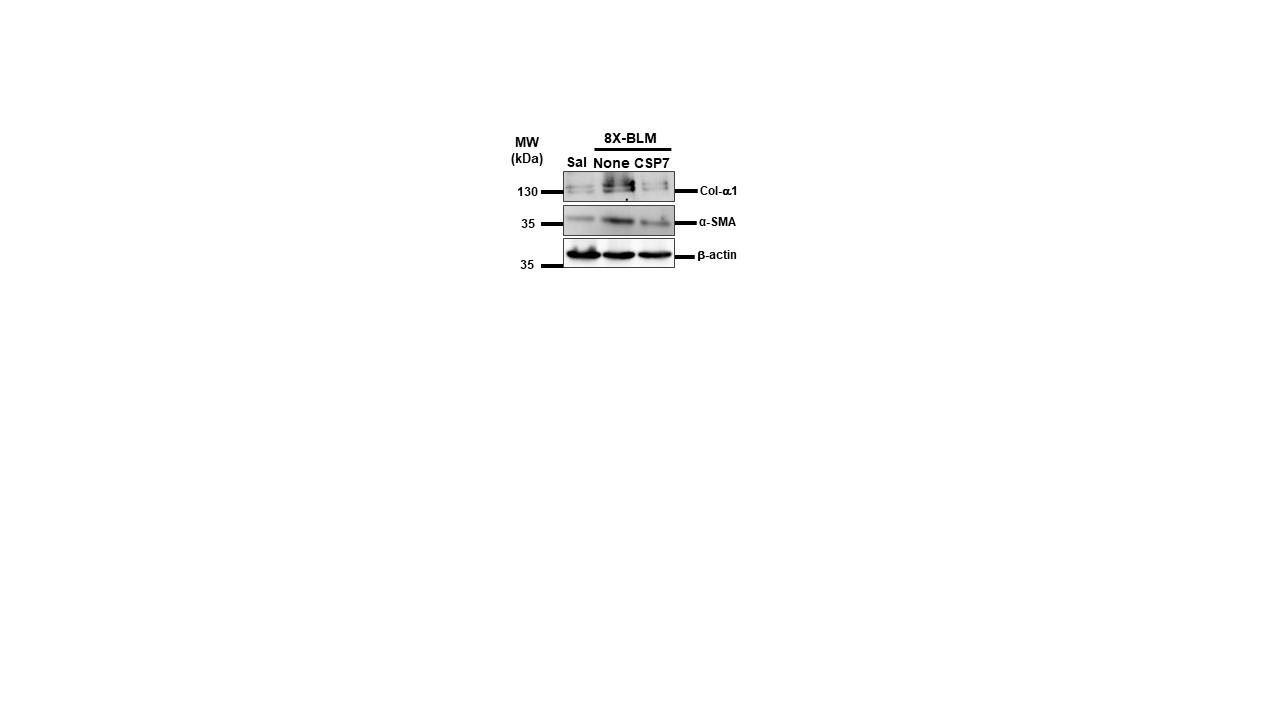


**Figure S3. Development of 8X-BLM-induced PF in WT mice.** Representative Western blot images showing the expression of Col1 and α-SMA in lung homogenates of saline or 8X-BLM or 8X-BLM+CSP7 treated WT mice. MW: Molecular weight. kDa: Kilodalton. The experiment was repeated at least 2 times with similar results.
